# The efficacy and safety of low-dose triple combination for hypertension treatment: a systematic review and meta-analysis of randomized controlled trials

**DOI:** 10.1007/s00210-025-03790-z

**Published:** 2025-02-06

**Authors:** Mohamed S. Elgendy, Hosam I. Taha, Ahmed Mazen Amin, Yehya Khlidj, Mohamed R. Ezz, Mohamed A. Elgamasy, Ahmed Almezaine, Mohamed A. Faheem, Islam Rajab, Mohamed Abuelazm

**Affiliations:** 1 Faculty of Medicine, Tanta University, Tanta, Egypt; 2 Faculty of Medicine, Mansoura University, Mansoura, Egypt; 3 Faculty of Medicine, Algiers University 1, Algiers, Algeria; 4 Internal Medicine Department, St Joseph University Medical Center, Paterson, NJ USA

**Keywords:** Blood pressure, Cardiovascular, Clinical trial, Anti-hypertensive, Single pill combination, Low-dose

## Abstract

Recently, Low-dose triple single-pill combinations (LDTC) have become a promising option for managing hypertension. This review evaluates LDTC’s effectiveness and safety versus standard care, monotherapy, and placebo for blood pressure (BP) control. A systematic review and meta-analysis of randomized controlled trials retrieved from PubMed, EMBASE, WOS, Scopus, and Cochrane from inception to September 2024. The analysis presented risk ratios (RR) for dichotomous outcomes and mean differences (MD) for continuous outcomes, with 95% confidence intervals (CI). PROSPERO ID: CRD42024595331. We identified five eligible trials with a total of 1,709 patients. LDTC had a higher rate of achieving BP < 140/90 at 4 to 6 weeks (wk) (RR: 1.56; CI: 1.41, 1.72; *p* < 0.01) and at 8 to 12 wk (RR: 1.43; CI: 1.31, 1.57; *P* < 0.01). Additionally, LDTC significantly reduced the automated office systolic BP at 4 to 6 wk (MD: -8.80; CI: -10.16, -7.44; *p* < 0.01), 8 to 12 wk (MD: -8.30; CI: -11.18, -5.42; *P* < 0.01), and at 24 wk (MD: -6.94; CI: -10.56, -3.32; *P* < 0.01). However, LDTC indicated an increased rate of hypokalemia (RR: 2.25; CI: 1.50, 3.38; *P* < 0.01), with no difference between both groups in total adverse events (AEs) (*P* = 0.44), serious AEs (*P* = 0.79), treatment discontinuation due to AEs (*P* = 0.91), and the AEs of special interest (*P* = 0.54). LDTC therapy is effective and safe for hypertension management but poses potassium depletion. Further large-scale studies are essential to confirm its clinical benefits.

**Graphical Abstract:**

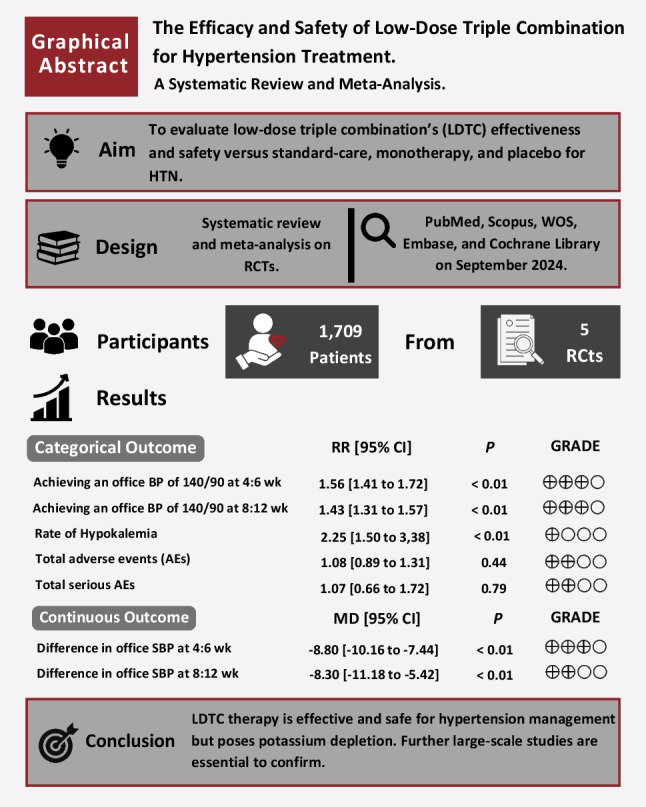

**Supplementary Information:**

The online version contains supplementary material available at 10.1007/s00210-025-03790-z.

## Introduction

Hypertension affects approximately one-third of the adult population aged 30–79 worldwide. Notably, 26% of the diagnosed cases have uncontrolled blood pressure (BP) levels that exceed the recommended target values (Organization WH 2023). In the US only, it is estimated that there are roughly 100.4 million adults with uncontrolled hypertension (Richardson et al. 2024). This population is at an increased risk of all-cause and cardiovascular disease mortality in comparison to patients with proper BP control (Zhou et al. 2018).

The initial therapeutic regimens with anti-hypertensive monotherapy plus lifestyle changes can be effective for achieving systolic BP (SBP) and/or diastolic BP (DBP targets) while reducing the risks of polypharmacy. However, a high proportion of individuals with uncontrolled hypertension are on monotherapy, which represents a missed opportunity to lower BP and cardiovascular risk. This can be due to patient-related factors (i.e., nonadherence) and treatment-related factors, notably the suboptimal efficacy of the monotherapy approach. (Wald et al. 2009; Zhang and Moran 2017) In this case, the escalation of the treatment aggressiveness is imposed either by increasing drug dosages or adding new drug classes (Unger et al. 2020). However, in most cases, combination therapy is considered at advanced hypertension stages, which explains why patients who receive ≥ 3 different classes of prescribed anti-hypertensive medications have worse SBP control and a greater risk of cardiovascular events (Zhang et al. 2024).

In light of this dilemma, a new strategy has been investigated in which triple anti-hypertensive therapy is started at lower doses (low-dose triple combination, LDTC) as the primary regimen instead of the standard care. Thus, several randomized controlled trials (RCTs), with some reaching phase III, have recently demonstrated the superiority of this approach over conventional single drug-based approach or placebo in treatment-naïve hypertension patients (Webster et al. 2018; Hong et al. 2020; Sung et al. 2022; Wang et al. 2023; Ojji et al. 2024; Rodgers et al. 2024b). In a study of 700 participants, LDTC was tested as an initial therapy for mild to moderate hypertension, given in the form of one pill containing 20 mg of telmisartan, 2.5 mg of amlodipine, and 12.5 mg of chlorthalidone. Remarkably, the treatment approach was associated with an increased proportion of patients with adequate BP values (Webster et al. 2018). In another phase II study, the same combination was found to induce a higher reduction in BP among patients with essential hypertension compared to placebo, with comparable safety outcomes (Ojji et al. 2024; Rodgers et al. 2024b).

Other relatively large RCTs have further supported the clinical benefit of LDTC-based regimens, suggesting that it could be an effective and well-tolerated option for the initial or early management of mild to moderate hypertension (Ojji et al. 2024; Rodgers et al. 2024b). The accumulation of this data has driven us to carry out the current systematic review and meta-analysis to assess the efficacy and safety of LDTC for hypertension.

## Methodology

### Protocol registration

This systematic review and meta-analysis were performed according to the PRISMA (Preferred Reporting Items for Systematic Reviews and Meta-Analyses) framework (Supplementary Table 1) (Page et al. 2021) and in accordance with the Cochrane Handbook for systematic reviews and meta-analyses (Higgins et al. 2024). The protocol of this review was registered in the PROSPERO database under the registry ID: CRD42024595331.

### Data sources and search strategy

Two authors (M.S.E. and H.I.T) carried out a systematic literature search independently across electronic databases: PubMed (MEDLINE), WOS, Scopus, Cochrane Library (CENTRAL), and EMBASE from inception to September 8th, 2024, without data and language filters. We combined free-text keywords and MeSH terms for ("Triple") AND ("Single Pill") AND ("hypertension"). M.S.E. and H.I.T. conducted reference screening and random search on Google Scholar and ResearchGate to avoid missing relevant articles. A detailed search is provided in Supplementary Table 2.

### Eligibility criteria

We included all clinical trials that aligned with our PICO criteria, as demonstrated in Table 1.

**Table 1:** Summary of our inclusion criteria (PICOS)

PICOS criteria	Description
Population (P):	Adult patients aged > 18 years with mild to moderate hypertension
Intervention (I):	Low-dose triple single-pill combination therapy consists of three antihypertensive agents selected from angiotensin II receptor blockers (ARBs), calcium channel blockers, and thiazide-like diuretics in various doses. Low-dose refers to the use of drugs at half, third, or quarter of the standard dose for hypertension only
Comparison (C):	Standard-care (which may include monotherapy or dual therapy) or placebo
Outcomes (O):	**The primary outcomes:** The proportion of patients achieving an automated office BP target of 140/90 mmHg at 4 to 6 weeks, 8 to 12 weeks, and 24 weeks, as well as achieving a home BP target of 130/80 mmHg at 4 weeks **The secondary outcomes:** Efficacy outcomes, including the difference in automated office SBP and DBP from baseline at 4 to 6 weeks, 8 to 12 weeks, and 24 weeks, while the difference in home SBP and DBP were measured at 4 weeks. Additionally, the safety outcomes include the incidence of any adverse events (AEs), serious adverse events (SAEs), and treatment discontinuation due to AEs and AEs of special interest (AESIs). The study additionally assessed drug-related and any AEs such as headache, dizziness, hypotension, and peripheral edema, as well as musculoskeletal pain, and abnormal laboratory findings included a decrease in estimated glomerular filtration rate (eGFR) of more than 30%, potassium levels below 3.5 or above 5.5 mmol/L, and sodium levels below 135 or above 145 mmol/L
Study design (S):	Parallel-group, randomized controlled trials

*BP* blood pressure; *SBP* systolic blood pressure; *DBP* diastolic blood pressure

The standard doses for the drugs under investigation are as follows: amlodipine (5 mg), telmisartan (40 mg), losartan (50 mg), chlorthalidone (12.5 mg), and indapamide (2.5 mg), based on recommended clinical guidelines by the WHO Collaborating Centre for Drug Statistics Methodology (https://www.whocc.no/atc_ddd_index/). The low dose is reduced relative to the standard, typically half, one-third, or a quarter of the standard dosage.

Additionally, we excluded animal studies, in vitro experiments on tissues and cultures, pilot and feasibility studies, cohort studies, case–control studies, cross-sectional studies, case series and reports, single-arm, cluster, crossover, and non-randomized trials, book chapters, editorials, press articles, reviews, theses, studies with incomplete or duplicate data, unpublished work or protocols without results, and conference abstracts. We also excluded studies utilizing triple multiple pills other than single-pill combinations.

### Study selection

Our review screening process was conducted using the Covidence online software, where duplicates were removed. Five independent reviewers (M.A.F., M.A.E., M.R.E., A.A., H.I.T) then screened the remaining records based on their titles and abstracts. Studies that met our inclusion criteria were further assessed through a full-text screening. In parallel, a sixth reviewer (M.S.E.) resolved any disagreements.

### Data extraction

Five reviewers (M.S.E., H.I.T., M.R.E., A.A., and M.A.E.), at least two independently for each section, extracted the data using a pre-designed sheet initially formatted through a pilot data extraction process on Excel (Microsoft, USA). The extracted data was organized into three key sections: (1) summary (study ID, study design, country, sample size, study arms, regimen, inclusion criteria, primary outcome, wash-out period, baseline BP, and maximum follow-up period). (2) baseline characteristics of patients: (e.g., demographic data, past medical history). (3) outcomes data for efficacy (the proportion of patients achieving an automated office BP target of 140/90 mmHg at 4 to 6 weeks, 8 to 12 weeks, and 24 weeks, achieving a home BP target of 130/80 mmHg at 4 weeks, change in automated office SBP and DBP from baseline at 4 to 6 weeks, 8 to 12 weeks, and 24 weeks, difference in home SBP and DBP at 4 weeks. The safety outcomes included (the incidence of any AEs, SAEs, treatment discontinuation due to AEs, AEs of special interests (AESIs), drug-related or any AEs such as (headache, hypotension, dizziness, peripheral edema, hypotension, and musculoskeletal pain), and abnormal laboratory findings).

We combined means and standard deviations (SDs) across dosage groups using weighted averages and pooled variance, as outlined in the Cochrane Handbook (Higgins et al. 2024), with a correlation coefficient 0.5. For dichotomous outcomes, events and sample sizes were summed to derive proportions.

### Risk of bias and certainty of evidence

The quality of the included RCTs was assessed using the Cochrane Risk of Bias 2 tool (Sterne et al. 2019) based on five domains: biases in randomization, deviations from the intended intervention, missing outcome data, outcome measurement deviations, and selective reporting of results. Then, these studies were categorized as having "low risk," "some concerns," or "high risk." This review was carried out by three reviewers (M.S.E., H.I.T., and M.R.E.), and disagreements were resolved by discussion with the fourth reviewer (M.A.). Publication bias was considered but could not be assessed as less than ten studies were included. (Sterne et al. 2019).

The certainty of the evidence was evaluated by M.S.E. using the GRADE system (Grading of Recommendations, Assessment, Development, and Evaluation) (Guyatt et al. 2008), which assesses five domains involving risk of bias, inconsistency, indirectness of evidence, imprecision, and other considerations (e.g., publication bias). The quality of evidence was rated to be high, moderate, low, or very low certainty. Finally, the grading was reviewed by M.A. to ensure transparency.

### Statistical analysis

We conducted the statistical analysis using R software version 4.3.1. For dichotomous outcomes, we used the risk ratio (RR), and for continuous outcomes, the mean difference (MD), both with 95% confidence intervals (CI). A random-effects model was applied when significant heterogeneity was detected (I^2^ > 50%), while a common-effect model was used when heterogeneity was not significant (I^2^ < 50%). Heterogeneity was evaluated using the chi-square test and the I-square statistic, where the chi-square test indicated the presence of heterogeneity and the I-square statistic measured its extent. We considered an alpha level of less than 0.1 for the chi-square test to denote significant heterogeneity. Additionally, we performed a sensitivity analysis by sequentially removing each study to identify potential sources of heterogeneity in the heterogenous outcomes. A subgroup analysis was also conducted to explore the impact of different dosages on the outcomes.

## Results

### Study selection

**Fig. 1:**
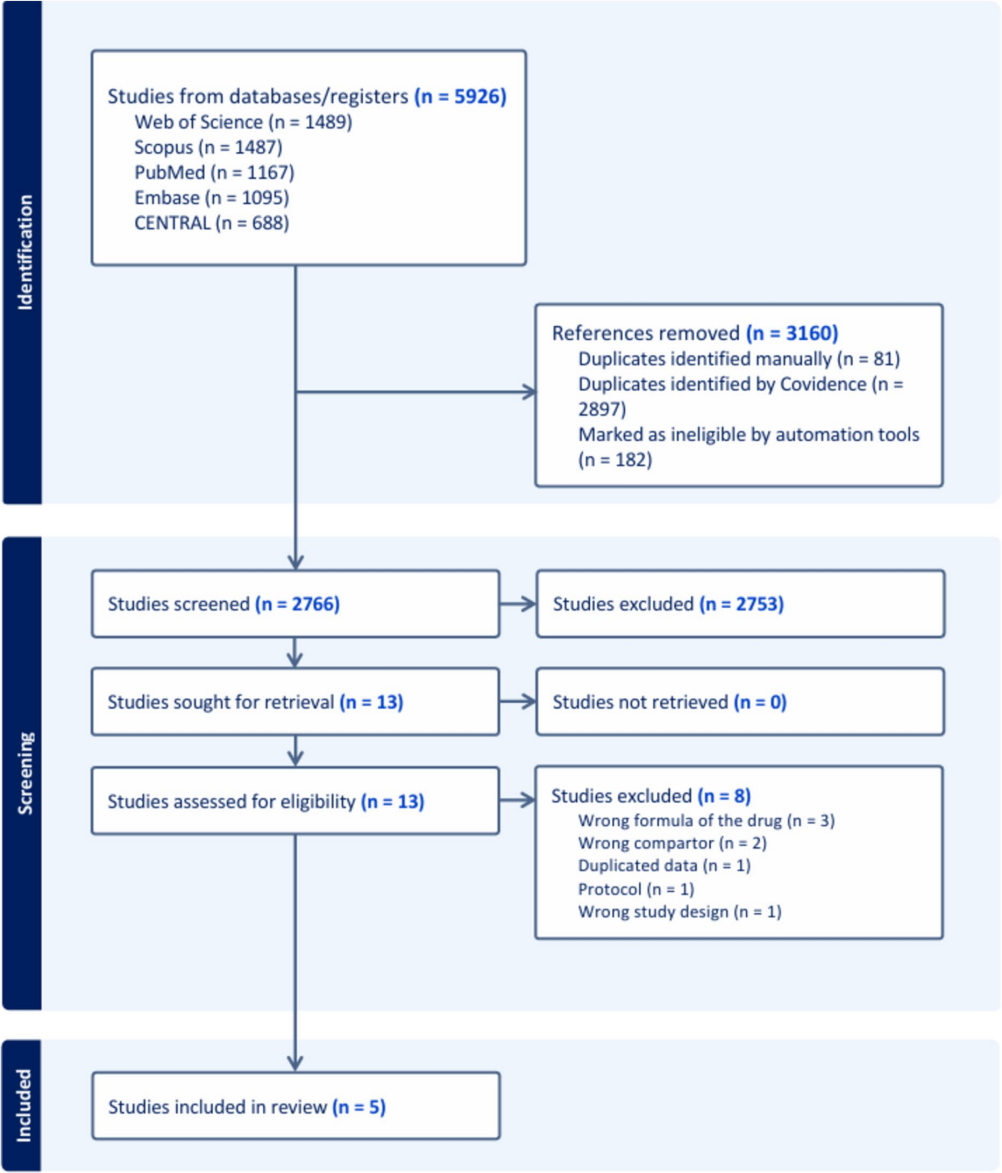
PRISMA flow chart for the systematic search and selection process

Our initial database search yielded 5926 records. After the deduplication and automation tool removal of 3160 by Covidence, 2766 unique studies remained for the title and abstract screening, and 2753 studies were excluded. The remaining 13 studies were selected for a thorough full-text review by a detailed assessment against predefined inclusion and exclusion criteria; eight studies were excluded (Punzi 2014; Mourad et al. 2017; Nedogoda and Stojanov 2017; Rakugi et al. 2018; Lung et al. 2019; Masharipov et al. 2020; Rhee et al. 2024), as detailed in Supplementary Table 3. Finally, we were left with five studies (Webster et al. 2018; Hong et al. 2020; Sung et al. 2022; Ojji et al. 2024; Rodgers et al. 2024b) that met all the criteria. The PRISMA flow diagram demonstrates the selection process (Fig. 1).

### Characteristics of included studies

We included five trials and 1,709 patients, with 910 in the LDTC group and 799 in the control group. The studies spanned diverse geographic regions, including the United States, the United Kingdom, Sri Lanka, Australia, Nigeria, and Korea. Three trials (Hong et al. 2020; Sung et al. 2022; Rodgers et al. 2024b) included a wash-out period, while the TRIUMPH trial (Webster et al. 2018) did not require it, and Ojji et al. 2024 discontinued monotherapy immediately before starting. All studies used a combination of three drugs as intervention selected from the following five: amlodipine, telmisartan, losartan, chlorthalidone, and indapamide. The administration regimen was in a fixed low dose except for Ojji et al. 2024, which employed an up-titration. The follow-up periods ranged from four to 24 weeks, averaging 13.6 weeks.

The target BP control was generally set at < 140/90 mmHg in the five included trials. However, Webster et al. 2018 applied a stricter target of < 130/80 mmHg only for the part of participants with diabetes or CKD, reflecting their higher cardiovascular risk. The other trials used the < 140/90 mmHg target for all participants without adjustments for high-risk groups. BP measurements for SBP and DBP outcomes were conducted across included trials using validated automated devices, ensuring consistency and reducing observer bias. Participants were seated and rested before multiple readings, with averages calculated for accuracy. Rodgers et al. 2024b also implemented secure data handling with direct transfer records into databases. A detailed summary of the included RCTs’ characteristics is provided in Table 2.

**Table 2:** Summary characteristics of the included trials

Study ID	Study design	Country	Total, *N*	Study arms		Regimen	Inclusion Criteria		Primary outcome	Wash-out	Baseline BP, mmHg	MFUP, W
				Intervention	Control		Condition	TTT Status				
Rodgers et al. 2024b	Multicenter, double-blind, RCT	USA, UK, Sri Lanka, Australia, Nigeria	295	GMRx2 (TEL, AML, ANDA.); * 1/2 comb (20; 2.5; 1.25 mg) * 1/4 comb (10; 1.25; 0.625 mg**)**	Placebo	Fixed	HTN with low calculated CV risk	Untreated or on mono	The change in home SBP from baseline to w 4	2 W	139/87	4
Ojji et al. 2024	Multicenter, open-label, RCT	Nigeria	300	GMRx2 (comb of TEL, AML, and ANDA.)	Standard-care protocol	Up-titration	Self-identified Black African with HTN	Untreated or on mono	The change in home SBP from baseline	Stopped mono if used	156/97	24
Sung et al. 2022	Multicenter, double-blind, RCT	Korea	176	TEL, AML, CHTD: * **1/4 comb:** (10; 1.25; 3.125 mg) * 1/3 comb: (13.3; 1.67; 4.167 mg) * 1/2 comb: (20; 2.5; 6.25 mg)	Mono with AML 5 mg & 10 mg; TEL 80 mg OR placebo	Fixed	HTN only or with Diabetic or chronic renal disease patients: 130 ≤ SBP < 180 mm Hg	Untreated or on therapy	The change in mean sitting SBP from baseline to w 8	2 W	151/92	8
Hong et al. 2020	Multicenter, double-blind, RCT	Korea	248	AML, LO, CHTD: * 1/2 comb (2.5; 25; 6.25 mg) * 1/3 comb (1.67; 16.67; 4.17 mg**)** * 1/4 comb (1.25; 12.5; 3.13 mg)	Mono with AML 5 mg & 10 mg, LO 100 mg, OR placebo	Fixed	HTN without severe heart/renal/hepatic disease	Untreated or on therapy	The change in the mean sitting SBP from baseline to w 8	1- to 2-W	154/92	8
Webster et al. 2018(TRIUMPH)	Multicenter, open-label, RCT	Sri Lanka	700	**1/2 comb:** TEL, 20 mg; AML, 2.5 mg; CHTD, 6.25 mg	Standard-care protocol	Fixed	Essential HTN with or without CKD or DM	Untreated or on mono	The proportion of achieving target BP at 6 months	No wash-out	154/90	24

*N* number; *TTT* treatment; *W* week; *MFUP* maximum follow-up period; *RCT* randomized controlled trial; *BP* blood pressure; *SBP* systolic blood pressure; *Comb* combination; *TEL* telmisartan; *AML* amlodipine; *INDA* indapamide; *CHTD* chlorthalidone; *Mono* monotherapy; *LO* losartan; *HTN* hypertension; *CKD* chronic kidney disease; *DM* diabetes mellitus

Gender distribution was balanced, with females present at 50.5% and males at 49.5% of participants, and the mean age across studies was 55.9 (± 11.56) years. The baseline SBP and DBP of included participants demonstrated mild-to-moderate hypertension, with a mean SBP of 151.3 mmHg (± 12.8) and a mean DBP of 90.7 mmHg (± 9.7) across the studies. A detailed summary of patient characteristics is provided in Supplementary Table 4 and Supplementary Table 5.

### Risk of bias and certainty of evidence

As demonstrated in Supplementary Fig. 1, All studies showcased a low risk of bias. Two studies were identified as open-label trials (Webster et al. 2018; Ojji et al. 2024); however, they used objective outcomes, robust methodology, and reliable measurement tools to help minimize the risk of performance and detection bias. Additionally, the summary of the GRADE assessment is presented in Table 3.

**Table 3:** Summary of GRADE evidence profile

Certainty assessment						№ of patients		Effect		Certainty
Studies	RoB	Inconsistency	Indirectness	Imprecision	Other	LDTC	Control	Relative (95% CI)	Absolute (95% CI)	
Achieving automated office BP 140/90 mmHg at 4 to 6 week										
(5) RCTs	not serious	not serious^a^	serious^b^	not serious	none	581/879 (66.1%)	330/786 (42.0%)	**RR 1.56** (1.41 to 1.72)	**235 more per 1,000** (from 172 to 302 more)	⨁⨁⨁◯ Moderate^a,b^
Achieving automated office BP 140/90 mmHg at 8 to 12 week										
(4) RCTs	not serious	not serious^a^	serious^b^	not serious	none	456/643 (70.9%)	349/716 (48.7%)	**RR 1.43** (1.31 to 1.57)	**210 more per 1,000** (from 151 to 278 more)	⨁⨁⨁◯ Moderate^a,b^
Difference in automated office SBP from baseline at 4 to 6 week										
(5) RCTs	not serious	not serious^a^	serious^b^	not serious	none	890	792	-	MD **8.8 mmHg lower** (10.16 lower to 7.44 lower)	⨁⨁⨁◯ Moderate^a,b^
Difference in automated office SBP from baseline at 8 to 12 week										
(4) RCTs	not serious	serious^c^	serious^b^	not serious	none	655	723	-	MD **8.3 mmHg lower** (11.18 lower to 5.42 lower)	⨁⨁◯◯ Low^b,c^
Any adverse events (AEs)										
(2) RCTs	not serious	not serious^a^	serious^d^	serious^e,f^	none	140/424 (33.0%)	133/450 (29.6%)	**RR 1.08** (0.89 to 1.31)	**24 more per 1,000** (from 33 fewer to 92 more)	⨁⨁◯◯ Low^a,d,e,f^
Potassium level < 3.5 mmol/L										
(3) RCTs	not serious	not serious^a^	serious^b^	very serious^d,g^	none	70/731 (9.6%)	28/564 (5.0%)	**RR 2.25** (1.50 to 3.38)	**62 more per 1,000** (from 25 to 118 more)	⨁◯◯◯ Very low^a,b,d,g^
Serious adverse events										
(5) RCTs	not serious	not serious^a^	serious^b^	serious^d,f^	none	32/905 (3.5%)	29/798 (3.6%)	**RR 1.07** (0.66 to 1.72)	**3 more per 1,000** (from 12 fewer to 26 more)	⨁⨁◯◯ Low^a,b,d,e^
Treatment discontinued due to AEs										
(5) RCTs	not serious	not serious^a^	serious^b^	serious^d,f^	none	31/905 (3.4%)	29/798 (3.6%)	**RR 0.97** (0.59 to 1.61)	**1 fewer per 1,000** (from 15 fewer to 22 more)	⨁⨁◯◯ Low^a,b,d,f^

*RoB* risk of bias; *LDTC* low-dose triple combination; *CI* confidence interval; *MD* mean difference; *RR* risk ratio; *RCT* randomized controlled trial; *BP* blood pressure; *SBP* systolic blood pressure; *DBP* diastolic blood pressure

**Explanations**

a. I^2 < 50%; shows no significant heterogeneity

b. Although the LDTC pill almost included the same drug classes, variations in dosing, treatment frequency, and duration across studies may have influenced clinical efficacy

c. I^2 > 50%; shows significant heterogeneity

d. A wide CI that does not exclude the appreciable risk of harm or benefit

e. Low number of events (< 300 if dichotomous) or a low number of patients (< 450 if continuous)

f. Crossing of no effect line (1), not excluding the risk of appreciable benefit/harm

g. Very wide CI that does not exclude the appreciable risk of harm or benefit

### Primary outcomes

#### Achieving target BP

In the five included trials, the target BP control was generally set at < 140/90 mmHg. However, Webster et al. 2018 applied a stricter target of < 130/80 mmHg only for the part of participants with diabetes or CKD, reflecting their higher cardiovascular risk. The other trials used the < 140/90 mmHg target for all participants without adjustments for high-risk groups.

**Fig. 2:**
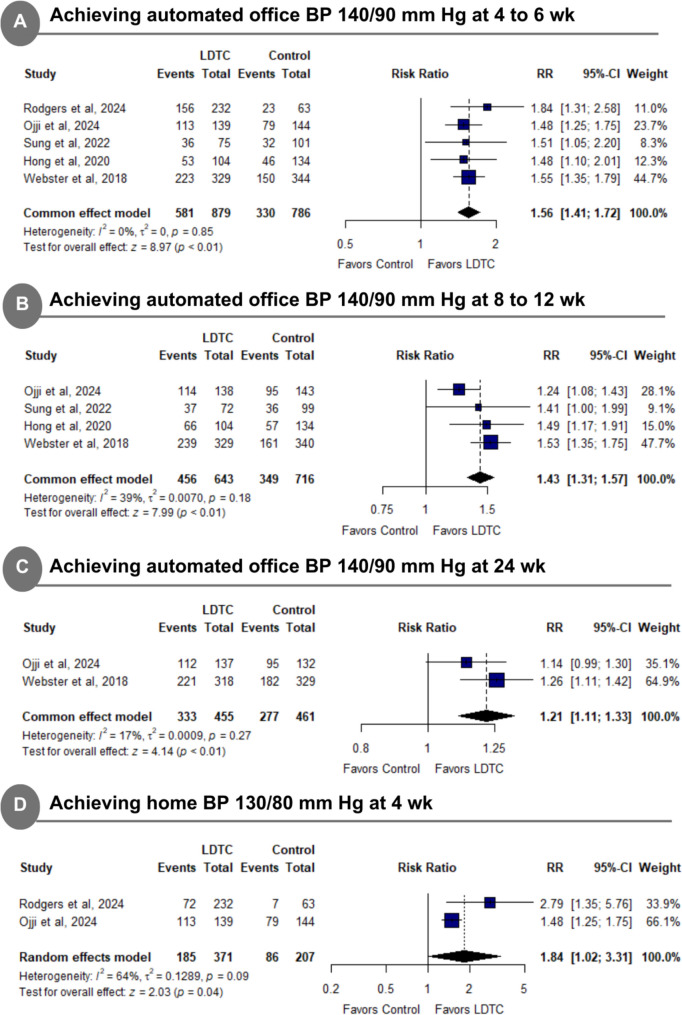
Forest plot of achieving target automated and home blood pressure. *BP* blood pressure, *LDTC* low-dose triple combination, *RR* risk ratio, *CI* confidence interval

LDTC was significantly associated with a higher incidence of achieving automated office target BP of 140–90 mmHg at 4 to 6 weeks (RR: 1.56 with 95% CI [1.41, 1.72], *P* < 0.01) (Fig. 2A), higher incidence of achieving automated office target BP of 40–90 mmHg at 8 to 12 weeks (RR: 1.43 with 95% CI [1.31, 1.57], *P* < 0.01) (Fig. 2B), higher incidence of achieving automated office target BP of 140–90 mmHg at 24 weeks (RR: 1.21 with 95% CI [1.11, 1.33], *P* < 0.01) (Fig. 2C), and higher incidence of achieving home target BP of 130–80 mmHg at 4 weeks (RR:1.84 with 95% CI [1.02, 3.31], *P* < 0.01) (Fig. 2D).

The pooled studies were homogenous in achieving an automated office target BP of 140–90 mmHg at 4 to 6 weeks (I^2^ = 0%, *P* = 0.85), achieving an automated office target BP of 140–90 mmHg at 8 to 12 weeks (I^2^ = 39%, *P* = 0.18), and achieving an automated office target BP of 140–90 mmHg at 24 weeks. However, pooled studies were heterogeneous in achieving a home target BP of 130–80 mmHg at 4 weeks (I^2^ = 64%, *P* = 0.09). Sensitivity analysis was not applicable. The test of subgroup difference according to the dosage was not significant in all primary outcomes (*P* > 0.1) (Supplementary Fig. 2).

### Secondary outcomes

#### Efficacy outcomes

##### Automated office BP measurements

BP measurements for SBP and DBP outcomes were conducted across included trials using validated automated devices, ensuring consistency and reducing observer bias. Participants were seated and rested prior to multiple readings, with averages calculated for accuracy.

**Fig. 3:**
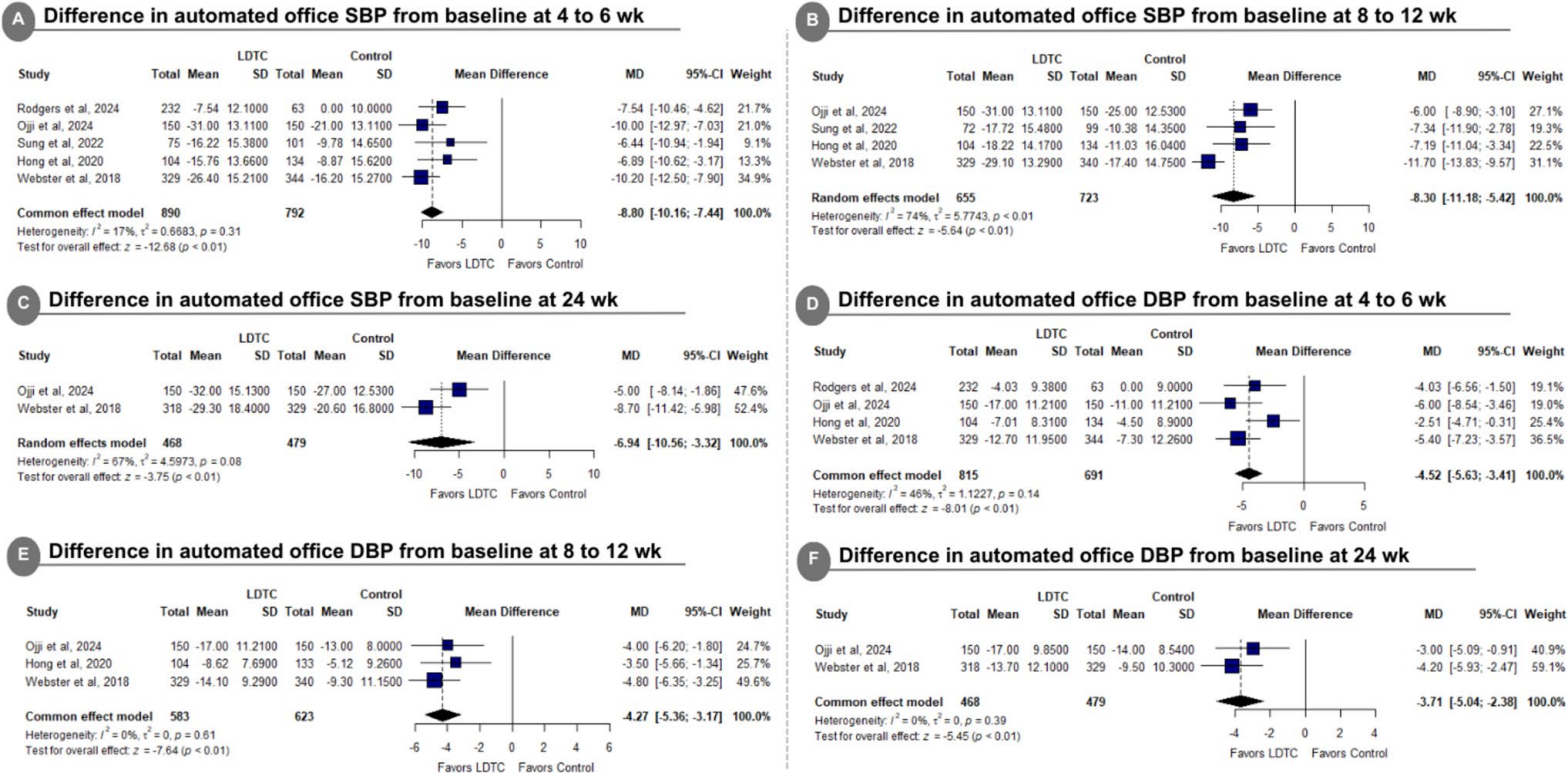
Forest plot of differences in automated office systolic and diastolic blood pressure from baseline. *SBP* systolic blood pressure, *LDTC* low-dose triple combination, *MD* mean difference, *CI* confidence interval, *DBP* diastolic blood pressure

LDTC was significantly associated with reduced automated office SBP from baseline at 4 to 6 weeks (MD: −8.80 with 95% CI [−10.16, −7.44], *P* < 0.01) (Fig. 3A), reduced automated office SBP from baseline at 8 to 12 weeks (MD: −8.30 with 95% CI [−11.18, −5.42], *P* < 0.01) (Fig. 3B), reduced automated office SBP from baseline at 24 weeks (MD: −6.94 with 95% CI [−10.56, −3.32], *P* < 0.01) (Fig. 3C), reduced automated office DBP from baseline at 4 to 6 weeks (MD: −4.52 with 95% CI [−5.63, −3.41], *P* < 0.01) (Fig. 3D), reduced automated office DBP from baseline at 8 to 12 weeks (MD: −4.27 with 95% CI [−5.36, −3.17], *P* < 0.01) (Fig. 3E), reduced automated office DBP from baseline at 24 weeks (MD: −3.71 with 95% CI [−5.04, −2.38], *P* < 0.01) (Fig. 3F), reduced home SBP from baseline at 4 weeks (MD: −7.00 with 95% CI [−8.95, −5.05], *P* < 0.01) (Supplementary Fig. 3A), and reduced home DBP from baseline at 4 weeks (MD: −4.76 with 95% CI [−6.42, −3.11], *P* < 0.01) (Supplementary Fig. 3B).

The pooled studies were homogenous in automated office SBP from baseline at 4 to 6 weeks (I^2^ = 17%, *P* = 0.31), automated office DBP from baseline at 4 to 6 weeks (I^2^ = 46%, *P* = 0.14), automated office DBP from baseline at 8 to 12 weeks (I^2^ = 0%, *P* = 0.61), automated office DBP from baseline at 24 weeks (I^2^ = 0%, *P* = 0.39), home SBP from baseline at 4 weeks (I^2^ = 0%, *P* = 1.00), and home DBP from baseline at 4 weeks (I^2^ = 0%, *P* = 0.76). However, pooled studies were heterogeneous in automated office SBP from baseline at 8 to 12 weeks (I^2^ = 74%, *P* < 0.01) and automated office SBP from baseline at 24 weeks (I^2^ = 67%, *P* = 0.08). For automated office SBP from baseline at 8 to 12 weeks, heterogeneity was best resolved after excluding TRIUMPH (Webster et al. 2018) (I^2^ = 0%) (Supplementary Fig. 4). Sensitivity analysis was not applicable for automated office SBP from baseline at 24 weeks.

**Fig. 4:**
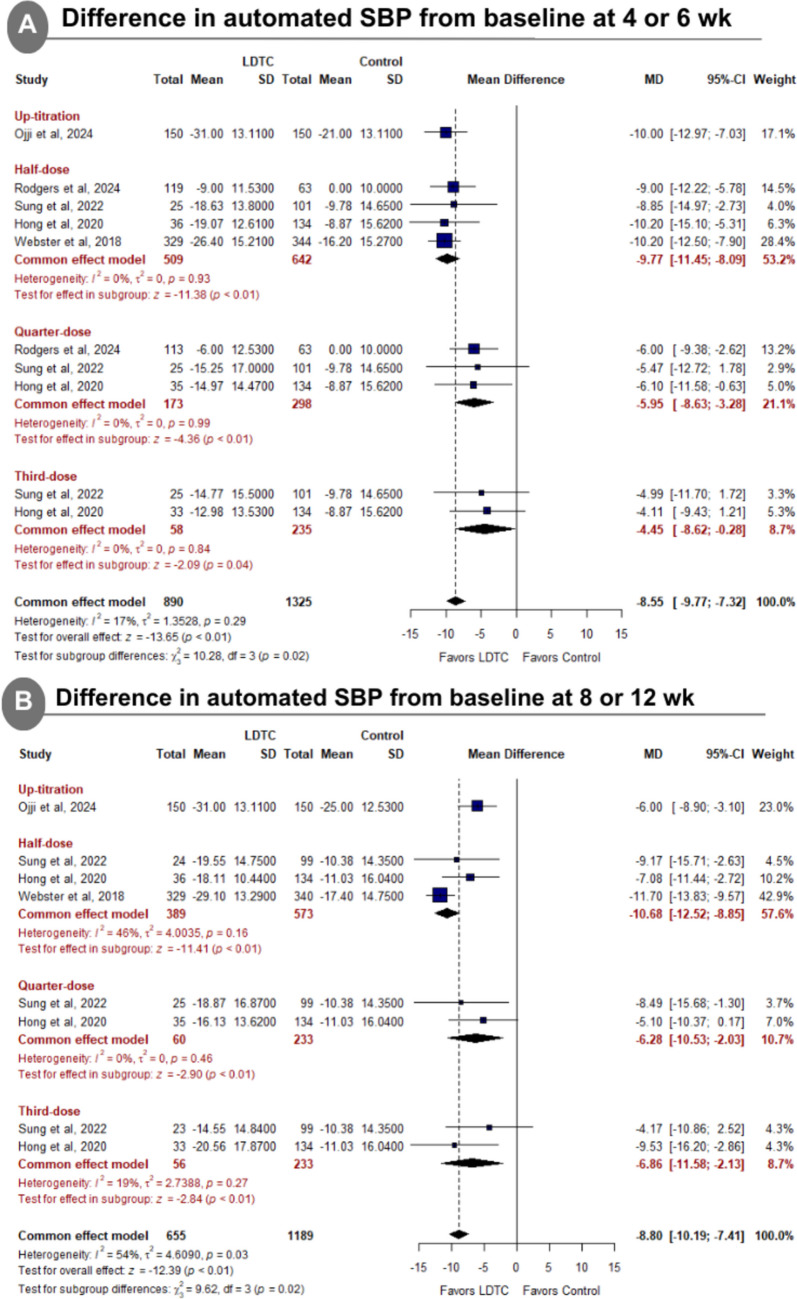
Forest plot of dosage subgroup analysis for difference in automated systolic blood pressure. *SBP* systolic blood pressure, *LDTC* low-dose triple combination, *MD* mean difference, *CI* confidence interval

Subgroup analysis showed statistical differences regarding the dosage for the difference in clinical SBP from baseline at 4 to 6 weeks (*P* = 0.02) (Fig. 4A), the difference in clinical SBP from baseline at 8 to 12 weeks (*P* = 0.02) (Fig. 4B), and the difference in clinical DBP from baseline at 4 to 6 weeks (*P* < 0.01) (Supplementary Fig. 5A). However, The test of subgroup difference according to the dosage was not significant in the difference in clinical DBP from baseline at 8 to 12 weeks (*P* = 0.83) (Supplementary Fig. 5B).

#### Safety outcomes

##### Drug-related and any AEs

All drug-related AEs are defined as events that led to the permanent discontinuation of the trial medication; however, any AEs are defined as overall AEs that occurred during the study period.

##### AEs of special interest (AESIs)

AESIs were identified as a combination of selected drug-related AEs that potentially led to treatment discontinuation, drawing from standardized definitions within Rodgers et al. 2024b and Ojji et al. 2024. Both calculated AESIs by combining the incidence of symptomatic hypotension, abnormal laboratory findings, headache, and peripheral edema.

##### Abnormal laboratory findings

Differences in the criteria for “all abnormal laboratory findings” were noted between studies. Rodgers et al. 2024b classified abnormal laboratory findings as any abnormalities in sodium, potassium, uric acid, glucose, lipids, creatinine, or eGFR. However, Ojji et al. 2024 defined abnormal laboratory findings as events judged to be clinically significant by site investigators, including abnormalities in sodium, potassium, uric acid, creatinine, or eGFR, without calculating glucose or lipid abnormalities.

**Fig. 5:**
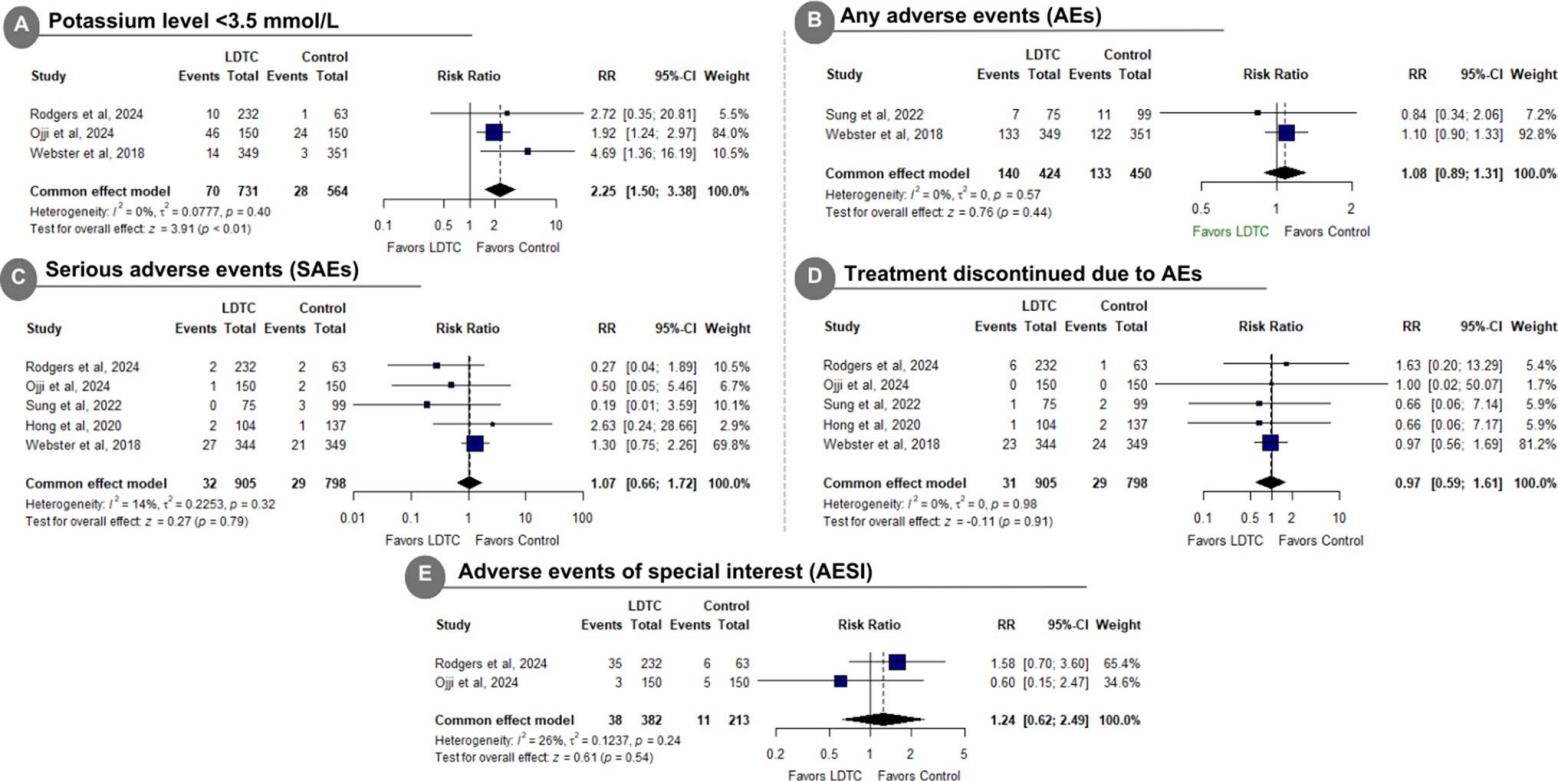
Forest plot of decreased potassium level, any adverse events (AEs), serious AEs, treatment discontinued due to AEs, and AEs of special interest. *LDTC* low-dose triple combination, *RR* risk ratio, *CI* confidence interval

LDTC was significantly associated with a higher incidence of hypokalemia (RR: 2.25 with 95% CI [1.50, 3.38], *P* < 0.01) (Fig. 5A). However, there was no statistically significant effect between LDTC and the control group in the incidence of total any AEs (RR: 1.08 with 95% CI [0.89, 1.31], *P* = 0.44) (Fig. 5B), any SAEs (RR: 1.07 with 95% CI [0.66, 1.72], *P* = 0.79) (Fig. 5C), treatment discontinuation due to AEs (RR: 0.97 with 95% CI [0.59, 1.61], *P* = 0.91) (Fig. 5D), AESIs (RR: 1.24 with 95% CI [0.62, 2.49], *P* = 0.54) (Fig. 5E), drug-related headache (RR: 0.56 with 95% CI [0.23, 1.36], *P* = 0.20) (Supplementary Fig. 6A), drug-related peripheral edema (RR: 1.18 with 95% CI [0.46, 3.00], *P* = 0.73) (Supplementary Fig. 6B), drug-related symptomatic hypotension (RR: 0.97 with 95% CI [0.36, 2.63], *P* = 0.96) (Supplementary Fig. 6C), drug-related dizziness (RR: 3.18 with 95% CI [0.77, 13.21], *P* = 0.11) (Supplementary Fig. 6D), any headache (RR: 1.13 with 95% CI [0.55, 2.32], *P* = 0.74) (Supplementary Fig. 7A), any hypotension (RR: 1.63 with 95% CI [0.60, 4.45], *P* = 0.34) (Supplementary Fig. 7B), any musculoskeletal pain (RR: 0.76 with 95% CI [0.45, 1.29], *P* = 0.31) (Supplementary Fig. 7C), all abnormal laboratory findings (RR: 2.60 with 95% CI [0.69, 9.81], *P* = 0.16) (Supplementary Fig. 8A), potassium > 5.5 mmol/L (RR: 1.29 with 95% CI [0.84, 1.99], *P* = 0.25) (Supplementary Fig. 8B), sodium < 135 mmol/L (RR: 2.10 with 95% CI [0.94, 4.71], *P* = 0.07) (Supplementary Fig. 8C), sodium > 145 mmol/L (RR: 0.87 with 95% CI [0.56, 1.34], *P* = 0.52) (Supplementary Fig. 8D), and eGFR decrease of > 30% (RR: 1.18 with 95% CI [0.79, 1.78], *P* = 0.42) (Supplementary Fig. 8E).

The pooled studies were homogenous in any AEs (I^2^ = 0%, *P* = 0.57), any SAEs (I^2^ = 14%, *P* = 0.32), treatment discontinuation due to AEs (I^2^ = 0%, *P* = 0.98), AESIs (I^2^ = 26%, *P* = 0.24), drug-related headache (I^2^ = 0%, *P* = 0.74), drug-related dizziness (I^2^ = 0%, *P* = 0.88), drug-related symptomatic hypotension (I^2^ = 23%, *P* = 0.28), drug-related peripheral edema (I^2^ = 0%, *P* = 0.91), any headache (I^2^ = 0%, *P* = 0.76), any hypotension (I^2^ = 0%, *P* = 0.58), any musculoskeletal pain (I^2^ = 0%, *P* = 0.87), all abnormal laboratory findings (I^2^ = 0%, *P* = 0.62), potassium < 3.5 mmol/L (I^2^ = 0%, *P* = 0.40), potassium > 5.5 mmol/L (I^2^ = 0%, *P* = 0.91), sodium < 135 mmol/L (I^2^ = 0%, *P* = 0.97), sodium > 145 mmol/L (I^2^ = 0%, *P* = 0.68), and eGFR decrease of > 30% (I^2^ = 34%, *P* = 0.22). Sub-group analysis regarding the dosage for all the safety outcomes was not significant (*P* > 0.1) (Supplementary Fig. 9–15).

### Discussion

The findings of the present systematic review and meta-analysis support, with a moderate level of certainty, the efficacy of LDTC in managing treatment-naïve hypertension patients or those on monotherapy compared to the standard first-line monotherapy or placebo. Hence, patients receiving LDTC therapy had a higher likelihood of achieving guidelines values of BP in the office setting at 24 weeks follow-up and in the home setting at 4 weeks follow-up. This group showed more significant SBP and DBP reductions from baseline values. Not surprisingly, a subgroup analysis revealed that the LDTC effect was dose-dependent. Concerning the safety analysis, there were higher odds of hypokalemia in the LDTC group.

In a previous meta-analysis, Wang et al. 2023 examined the data from seven randomized trials (*n* = 1,918 patients) comparing low-dose triple and quadruple combination pills to monotherapy, standard-care, or placebo for treatment-naïve hypertension individuals. Consistently with our findings, the authors reported a higher mean decrease in SBP and a more significant proportion of BP below 140/90 mm Hg at 4 to 12 weeks in the group receiving combination-based medication. This group showed no increase in the risk of treatment withdrawal or adverse events occurrence except for dizziness (Wang et al. 2023). Similarly, Abuelazm et al. 2023a demonstrated the effectiveness of the "quadpill," a single pill containing quarter doses of four anti-hypertensives, as a feasible and potent option for reducing BP (Abuelazm et al. 2023a).

The main difference with our study from Wang et al. 2023 was the inclusion of both triple and quadruple anti-hypertensive therapy as the interventional strategy. Furthermore, following this 2023 meta-analysis, two new RCTs with large sample sizes and a new combination of triple therapy were published (Rodgers et al. 2024b and Ojji et al. 2024); thereby, an updated examination of the evidence for LDTC alone was needed. Additionally, we focused exclusively on triple anti-hypertensive combinations, excluding studies involving quadruple combinations or any additional agents. We also specifically included only parallel-group RCTs, excluding crossover and cluster RCTs, following the recommendations of the Cochrane Handbook (Chapter 23) (Higgins et al. 2024). Notably, the Wald et al. 2012 trial, a crossover study incorporating simvastatin, was excluded to align with our PICO criteria and to ensure methodological consistency (Wald et al. 2012). Finally, unlike Wang et al. 2023, we conducted a quality assessment using the Cochrane RoB2 tool and performed the GRADE assessment.

The LDTC is designed to provide the maximum adequate control of hypertension without increasing the risk of AEs or SAEs. This can be achieved due to several advantages of LDTC. First, it is a more potent BP-lowering action. The LDTC included angiotensin II receptor blockers (ARBs, telmisartan or losartan), calcium channel blockers (CCBs, amlodipine), and diuretics (chlorthalidone or indapamide). Each of these classes acts primarily but not exclusively on a BP parameter, with ARBs mainly modulating the renin–angiotensin–aldosterone system (RAAS), CCBs reducing vessel tonus independently of RAAS, and diuretics lowering preload independent of vascular tonus and RAAS. Their combination would, therefore, ensure simultaneous targeting of different pathophysiological pathways, which results in additive anti-hypertensive effects, notably explaining the more significant reduction and adequate control in both SBP and DBP, whether at home or office measurements (Rodgers et al. 2024a). Notably, the combination of anti-hypertensive drugs was demonstrated to allow an extra BP reduction of approximately 5 times greater than when the dose of hypertension monotherapy is doubled (Wald et al. 2009).

Second, on a pharmacokinetic level, LDTC has the advantage of providing prolonged and sustained control of BP, which is due to the higher half-life of the used molecules. Hence, among ARBs, telmisartan is preferred since it is the longest-acting molecule in this class, with a half-life reaching 24 h, propriety driven by the drug’s very high lipophilicity (Wienen et al. 2000). Likewise, amlodipine is a long-acting CCB with a half-life of 35 h (for 5 mg), which is increased to 48 h in the elderly (Abernethy 1989). The average half-life of thiazide-like chlorthalidone is, on average, 42 h (much longer than hydrochlorothiazide) (Carter et al. 2004), whereas that of indapamide is about 16 h (Carter et al. 2004).

Third, the low doses in the triple combinations allow for the reduction of BP control aggressiveness, consequently lowering the risks of adverse events while reaching the recommended BP targets. Chlorthalidone at a low dose (6.25 mg) effectively reduces mean 24-h BP as well as daytime and night-time BP, yet it is more potent than hydrochlorothiazide (Pareek et al. 2016; Abuelazm et al. 2023b). Similar results are seen with low-dose indapamide (Inaba et al. 2004). When added to ARBs or CCBs monotherapy, the latter enhanced the anti-hypertensive efficacy without inducing any additional metabolic side effects (Yamada et al. 2010).

Hypokalaemia is an expected adverse event of diuretics with potassium-lowering effects, including the thiazide-like agents chlorthalidone or indapamide (Jin et al. 2018). Combining anti-hypertensive agents increases substantially the risk of hypokalemia as each class may alter the potassium homeostasis by a different mechanism. For instance, while thiazides (and thiazides-like) favor potassium excretion by kidneys, CCBs increase potassium loss by extra-renal pathways (Krogager et al. 2020). Future investigations may identify patients at risk of LDTC-induced hypokalemia, as this is crucial for implementing prevention strategies (i.e., potassium supplementation) and close monitoring. It would also be necessary to search for the combinations that result in the highest changes in potassium levels, thereby avoiding them in at-risk individuals. Concerns regarding this adverse event impose a more extensive exploration of the safety profile of LDTC.

### Limitations

The main limitations were as follows: (i) The relatively small number of the included studies would have affected the robustness of the examined data. (ii) The presence of heterogeneities, particularly for the outcome changes in automated office SBP, is likely the result of differences in the patient's baseline, LDTC regimen, and follow-up durations. (iii) A subgroup analysis regarding the most effective and tolerable triple combo of anti-hypertensive medications, although needed, was not possible due to data incompleteness. (iv) The lack of an extensive follow-up did not allow for comparing the long-term cardiovascular events between the LDTC group and controls, which is paramount for accurately evaluating the benefit of hypertension therapy (Norrie 2023). (v) Although the LDTC pill included the same drug classes across the included studies, the doses were not consistent, ranging from half, third, to quarter doses, with discrepancies in treatment frequency and duration. As a result, we cannot rule out the potential impact of these differing treatment regimens on clinical efficacy. (vi) The included trials are hindered by substantial heterogeneity in their control groups, which varied from placebo to monotherapy and standard care, introducing variability that may influence the comparability and interpretation of results.

### Implications for clinical practice & future research

Earlier BP control has important prognostic implications in patients with new-onset essential hypertension (Volpe et al. 2018). Hence, failed control of BP within the first year of hypertension diagnosis is associated with a higher likelihood of major cardiovascular events in the short term (O’Connor et al. 2013). The current guidelines recommend initiating pharmacological treatment in hypertension patients, typically with low-dose ACE inhibitors/ARBs and/or dihydropyridine/CCBs as a first step, then full dose as a second step, and finally, adding diuretics (Unger et al. 2020). Although our results provide a preliminary assessment of LDTC’s potential advantages that may qualify it to substitute current monotherapy or dual-therapy-based regimens, some questions are yet to be answered. It is unclear which population should benefit from primary LDTC and at which disease stage.

Hence, the patients included in the RCTs had different levels of cardiovascular risk (some with diabetes or renal disease, some without, others of black ethnicity, etc.). Moreover, the stages of hypertension varied across the trials (from grade 1 to grade 2), which creates an ambiguity regarding the optimal indication of LDTC initiation according to baseline BP values. Another significant unanswered question concerns the most effective and safe triple combination in a general sense or per hypertension group. Consequently, before any clinical use, more research is needed to provide a more extensive evaluation of LDTC benefits and risk balance among hypertension patients. Future studies should also aim to identify the best situation(s) where LDTC would be the most indicated and beneficial.

## Conclusion

The current RCTs-based evidence suggests that novel LDTC therapy could be more effective than standard care for hypertension management in patients who are untreated or receiving a single anti-hypertensive drug. Also, LDTC demonstrates a generally safe profile, except for an increased risk of hypokalemia; therefore, its use may require more caution and monitoring. Although such an approach appears to be promising in enabling an early achievement of BP targets, more large-scale studies comparing different dosages and drug regimens are still required.

## Supplementary Information

Below is the link to the electronic supplementary material.

Supplementary file1 (PDF 3274 KB)

## Data Availability

All source data for this work (or generated in this study) are available upon reasonable request.
