# Supplementary material for: The efficacy and safety of low-dose triple combination for hypertension treatment: a systematic review and meta-analysis of randomized controlled trials
Source: Naunyn Schmiedebergs Arch Pharmacol. 2025 Feb 6;398(7):7721–36. doi: 10.1007/s00210-025-03790-z (PMC12263486; doi:10.1007/s00210-025-03790-z)
Supplement: Supplementary file 1 — Supplementary file1 (PDF 3274 KB) [file 210_2025_3790_MOESM1_ESM.pdf]

Naunyn-Schmiedeberg's Archives of Pharmacology

Supplementary Material

Title.

The Efficacy and Safety of Low-Dose Triple Combination for Hypertension: A Systematic Review and Meta-Analysis of Randomized Controlled Trials.

Authors.

Mohamed S. Elgendy[†], Hosam I. Taha[†], Ahmed Mazen Amin, Yehya Khlidj, Mohamed R. Ezz, Mohamed A. Elgamasy, Ahmed Almezaine, Mohamed A. Faheem, Islam Rajab, Mohamed Abuelazm.

[†] These authors have contributed equally to this work and share the first authorship.

The authors have provided this supplementary material to demonstrate additional information about this study.

Index.

Supplementary Content:		
**a. Tables:**		**Page**
1	**Supplementary Table 1** PRISMA 2020 checklist.	4
2	**Supplementary Table 2** Search strategy and literature search results.	7
3	**Supplementary Table 3** List of excluded studies with reasons during the full-text screening process.	8
4	**Supplementary Table 4** Baseline characteristics of the included patients.	9
5	**Supplemental Table 5** Additional Baseline characteristics of the included patients.	10
**b. Figure:**		**Page**
1	**Supplementary Fig. 1** Overview of the risk of bias of the included randomized controlled trials.	11
2	**Supplementary Fig. 2** Forest plot of dosage subgroup analysis for achieving target automated office blood pressure control.	12
3	**Supplementary Fig. 3** Forest plot of difference in home systolic and diastolic blood pressure at 4 weeks.	13
4	**Supplementary Fig. 4** Leave-one-out sensitivity analysis of automated office systolic blood pressure from baseline at 8 to 12 weeks.	14
5	**Supplementary Fig. 5** Forest plot of dosage subgroup analysis for difference in automated diastolic blood pressure.	15
6	**Supplementary Fig. 6** Forest plot of drug-related adverse events (headache, peripheral edema, symptomatic hypotension, and dizziness).	16
7	**Supplementary Fig. 7** Forest plot of adverse effects for any reason. Any defined as the adverse event occurred in the patients for any reason across the study period.	17
8	**Supplementary Fig. 8** Forest plot of abnormal laboratory findings.	18
9	**Supplementary Fig. 9** Forest plot of dosage subgroup analysis for adverse events and serious adverse events.	19
10	**Supplementary Fig. 10** Forest plot of dosage subgroup analysis for treatment discontinuation due to adverse events.	20

**11**	**Supplementary Fig. 11** Forest plot of dosage subgroup analysis for drug-related adverse events (headache and dizziness).	**21**
**12**	**Supplementary Fig. 12** Forest plot of dosage subgroup analysis for drug-related adverse events (peripheral edema and hypotension).	**22**
**13**	**Supplementary Fig. 13** Forest plot of dosage subgroup analysis for adverse effects for any reason.	**23**
**14**	**Supplementary Fig. 14** Forest plot of dosage subgroup analysis for decreased and increased potassium levels.	**24**
**15**	**Supplementary Fig.15** Forest plot of dosage subgroup analysis for decreased and increased sodium levels and decreased estimated glomerular filtration rate.	**25**

Supplementary Table 1 PRISMA 2020 checklist.

Section and Topic	Item #	Checklist item	Location where item is reported
**TITLE**			
Title	1	Identify the report as a systematic review.	Line 3, Page 1
**ABSTRACT**			
Abstract	2	See the PRISMA 2020 for Abstracts checklist.	Page 2
**INTRODUCTION**			
Rationale	3	Describe the rationale for the review in the context of existing knowledge.	Page 4
Objectives	4	Provide an explicit statement of the objective(s) or question(s) the review addresses.	Page 4, 5
**METHODS**			
Eligibility criteria	5	Specify the inclusion and exclusion criteria for the review and how studies were grouped for the syntheses.	Table 1, page 6 subsection 2.3
Information sources	6	Specify all databases, registers, websites, organisations, reference lists and other sources searched or consulted to identify studies. Specify the date when each source was last searched or consulted.	Page 6, subsection 2.2
Search strategy	7	Present the full search strategies for all databases, registers and websites, including any filters and limits used.	Supplementary material, table S2
Selection process	8	Specify the methods used to decide whether a study met the inclusion criteria of the review, including how many reviewers screened each record and each report retrieved, whether they worked independently, and if applicable, details of automation tools used in the process.	Page 7, subsection 2.4
Data collection process	9	Specify the methods used to collect data from reports, including how many reviewers collected data from each report, whether they worked independently, any processes for obtaining or confirming data from study investigators, and if applicable, details of automation tools used in the process.	Page 7, 8, subsection 2.5
Data items	10a	List and define all outcomes for which data were sought. Specify whether all results that were compatible with each outcome domain in each study were sought (e.g. for all measures, time points, analyses), and if not, the methods used to decide which results to collect.	Page 7, subsection 2.5
	10b	List and define all other variables for which data were sought (e.g. participant and intervention characteristics, funding sources). Describe any assumptions made about any missing or unclear information.	Page 7, 8, subsection 2.5
Study risk of bias assessment	11	Specify the methods used to assess risk of bias in the included studies, including details of the tool(s) used, how many reviewers assessed each study and whether they worked independently, and if applicable, details of automation tools used in the process.	Page 8, subsection 2.6
Effect measures	12	Specify for each outcome the effect measure(s) (e.g. risk ratio, mean difference) used in the synthesis or presentation of results.	Page 9, subsection 2.7
Synthesis methods	13a	Describe the processes used to decide which studies were eligible for each synthesis (e.g. tabulating the study intervention characteristics and comparing against the planned groups for each synthesis (item #5)).	Page 8, 9, subsection 2.7
	13b	Describe any methods required to prepare the data for presentation or synthesis, such as handling of missing summary statistics, or data conversions.	Page 9, subsection 2.7
	13c	Describe any methods used to tabulate or visually display results of individual studies and syntheses.	Page 9, subsection 2.7
	13d	Describe any methods used to synthesize results and provide a rationale for the choice(s). If meta-analysis was performed, describe the model(s), method(s) to identify the presence and extent of statistical heterogeneity, and software package(s) used.	Page 9, subsection 2.7
	13e	Describe any methods used to explore possible causes of heterogeneity among study results (e.g. subgroup analysis, meta-regression).	Page 9, subsection 2.7

Section and Topic	Item #	Checklist item	Location where item is reported
	13f	Describe any sensitivity analyses conducted to assess robustness of the synthesized results.	Page 8, subsection 2.6
Reporting bias assessment	14	Describe any methods used to assess risk of bias due to missing results in a synthesis (arising from reporting biases).	Page 9, subsection 2.6
Certainty assessment	15	Describe any methods used to assess certainty (or confidence) in the body of evidence for an outcome.	Page 8, subsection 2.6
**RESULTS**			
Study selection	16a	Describe the results of the search and selection process, from the number of records identified in the search to the number of studies included in the review, ideally using a flow diagram.	Page 10, subsection 3.1
	16b	Cite studies that might appear to meet the inclusion criteria, but which were excluded, and explain why they were excluded.	Page 10, subsection 3.1
Study characteristics	17	Cite each included study and present its characteristics.	Page 10, subsection 3.2
Risk of bias in studies	18	Present assessments of risk of bias for each included study.	Page 11, subsection 3.3
Results of individual studies	19	For all outcomes, present, for each study: (a) summary statistics for each group (where appropriate) and (b) an effect estimate and its precision (e.g. confidence/credible interval), ideally using structured tables or plots.	Pages 11-15, subsections 3.4-3.5
Results of syntheses	20a	For each synthesis, briefly summarise the characteristics and risk of bias among contributing studies.	Page 11, subsection 3.3
	20b	Present results of all statistical syntheses conducted. If meta-analysis was done, present for each the summary estimate and its precision (e.g. confidence/credible interval) and measures of statistical heterogeneity. If comparing groups, describe the direction of the effect.	Pages 11-15, subsections 3.4-3.5
	20c	Present results of all investigations of possible causes of heterogeneity among study results.	Pages 11-145 subsections 3.4-3.5
	20d	Present results of all sensitivity analyses conducted to assess the robustness of the synthesized results.	Not applicable
Reporting biases	21	Present assessments of risk of bias due to missing results (arising from reporting biases) for each synthesis assessed.	Page 11, subsection 3.3
Certainty of evidence	22	Present assessments of certainty (or confidence) in the body of evidence for each outcome assessed.	Page 11, subsection 3.3<br>Sup material, table S5
**DISCUSSION**			
Discussion	23a	Provide a general interpretation of the results in the context of other evidence.	Page 17
	23b	Discuss any limitations of the evidence included in the review.	Page 19, 20
	23c	Discuss any limitations of the review processes used.	Page 20
	23d	Discuss implications of the results for practice, policy, and future research.	Page 20
**OTHER INFORMATION**			
Registration and protocol	24a	Provide registration information for the review, including register name and registration number, or state that the review was not registered.	Page 6, subsection 2.1
	24b	Indicate where the review protocol can be accessed, or state that a protocol was not prepared.	Page 6, subsection 2.1
	24c	Describe and explain any amendments to information provided at registration or in the protocol.	Page 6, subsection 2.1
Support	25	Describe sources of financial or non-financial support for the review, and the role of the funders or sponsors in the review.	Page 22

Section and Topic	Item #	Checklist item	Location where item is reported
Competing interests	26	Declare any competing interests of review authors.	Page 22
Availability of data, code and other materials	27	Report which of the following are publicly available and where they can be found; template data collection forms; data extracted from included studies; data used for all analyses; analytic code; any other materials used in the review.	Page 22

Supplementary Table 2 Search strategy and literature search results.

<table>
<tr><th>Database</th><th>Restrictions</th><th>Access date</th><th>Search strategy</th><th>No of results</th></tr>
<tr><td>Pubmed</td><td>Title/abstract</td><td>05/09/2024</td><td rowspan="4">((Triple-therapy OR Triple-Pill OR Triple-medication OR Triple-combination) OR ((Triple) AND (antihypertensive* OR therapy OR drug OR medication OR Pill OR "Fixed combination" OR "fixed-dose combination" OR "Single-Pill" OR combination)))
AND ("blood pressure lowering" OR "high blood pressure" OR hypertension OR "blood pressure control")</td><td>1167</td></tr>
<tr><td>Cochrane</td><td>Title Abstract Keyword</td><td>05/09/2024</td><td>688</td></tr>
<tr><td>Scopus</td><td>Article title OR abstract</td><td>05/09/2024</td><td>1487</td></tr>
<tr><td>WOS</td><td>Topic</td><td>05/09/2024</td><td>1489</td></tr>
<tr><td>Embase</td><td>Title</td><td>08/09/2024</td><td>(((Triple-therapy) OR (Triple-Pill) OR (Triple-medication) OR (Triple-combination)) OR
((Triple) AND ((antihypertensive*) OR therapy OR drug OR medication OR Pill
OR (Fixed combination) OR (fixed-dose combination) OR (Single-Pill) OR (Single Pill) OR combination))) AND ((blood pressure) OR hypertension)</td><td>1095</td></tr>
<tr><td colspan="4">Total</td><td>5926</td></tr>
</table>

Supplementary Table 3 List of excluded studies with reasons during the full-text screening process.

Study ID	Title	Reason of exclusion
**Rhee et al., 2024**	SINGLE-PILL COMBINATION OF THIRD-DOSE TRIPLE ANTIHYPERTENSIVE THERAPY VERSUS STANDARD-DOSE MONOTHERAPY IN PATIENTS WITH MILD TO MODERATE HYPERTENSION	Does not meet our criteria - conference abstract
**Kim et al., 2023**	EFFICACY AND SAFETY OF SINGLE-PILL COMBINATION OF OLOMAXÂ (OLMESARTAN, AMLODIPINE, AND ROSUVASTATIN) IN HYPERTENSIVE PATIENTS WITH LOW-TOMODERATE CARDIOVASCULAR RISK	Does not meet our criteria - wrong comparator
**Masharipov et al., 2020**	The effectiveness of triple fixed-dose combination therapy in the management of uncontrolled arterial hypertension	Does not meet the criteria - wrong study design (prospective cohort)
**Lung et al., 2019**	Fixed-combination, low-dose, triple-pill antihypertensive medication versus usual care in patients with mild-to-moderate hypertension in Sri Lanka: a within-trial and modelled economic evaluation of the TRIUMPH trial	Does not meet our criteria - duplicated data
**Rakugi et al., 2018**	Effects of triple combination therapy with azilsartan/amlodipine/hydrochlorothiazide on office/home blood pressure: a randomized-controlled trial in Japanese essential hypertensive patients	Does not meet our criteria - wrong formula of the drug
**Nedogoda et al., 2017**	Single-Pill Combination of Perindopril/Indapamide/Amlodipine in Patients with Uncontrolled Hypertension: A Randomized Controlled Trial	Does not meet the criteria - wrong comparator
**Mourad et al., 2017**	Blood pressure-lowering efficacy and safety of perindopril/indapamide/amlodipine single-pill combination in patients with uncontrolled essential hypertension: a multicenter, randomized, double-blind, controlled trial	Does not meet the criteria - wrong comparator
**Punzi., 2014**	Efficacy and safety of olmesartan/amlodipine/hydrochlorothiazide in patients with hypertension not at goal with mono, dual or triple drug therapy: results of the CHAMPiOn study	Does not meet our criteria - wrong formula of the drug

Supplementary Table 4 Baseline characteristics of the included patients.

Study ID	Groups	No. of patients	Age, y, mean (SD)	Gender; Female, No. (%)	Current smoker, No. (%)	BMI, kg/m2, mean (SD)	Alcohol consumption, No. (%)	One BP lowering TTT, No. (%)	Heart rate, beats/min, mean (SD)	Diabetes type 2, No. (%)	CKD, No. (%)
**Rodgers et al. 2024**	**LDTC**	232	50.5 ± 11	129 (55.6)	20 (8.6)	30.64 ± 6.63	88 (37.9)	132 (45)	NM	18 (8)	NM
	**Control**	63	51 ± 13	36 (57)	4 (6)	30 ± 5.7	25 (40)	33 (52)	NM	3 (5)	NM
**Ojji et al. 2024**	**LDTC**	150	52 ± 10	81 (54)	1 (1)	28 ± 5	11 (7)	58 (39)	80 ± 14	2 (1.3)	0 (0)
	**Control**	150	51 ± 9	81 (54)	1 (1)	29 ± 6	11 (7)	57 (38)	79 ± 13	6 (4)	0 (0)
**Sung et al. 2022**	**LDTC**	75	62.3 ± 8.8	30 (40)	13 (17.3)	25.76 ± 3.64	NM	69 (92)	75.1 ± 11.2	21 (28)	0 (0)
	**Control**	101	59.9 ± 10.7	31 (30.7)	20 (19.8)	26.06 ± 3.55	NM	89 (88.1)	74.7 ± 12.5	19 (18.8)	0 (0)
**Hong et al. 2020**	**LDTC**	104	62.65 ± 9.9	29 (27.9)	20 (19.2)	25.68 ± 3.48	38 (55.8)	NM	NM	28 (27)	NM
	**Control**	134	64 ± 11.6	43 (32.1)	21 (15.6)	25.15 ± 3.06	67 (50)	NM	NM	32 (23.9)	NM
**Webster et al. 2018 (TRIUMPH)**	**LDTC**	349	56.4 ± 11.3	207 (59.3)	39 (11.2)	NM	42 (12)	140 (40.1)	78.1 ± 12.5	111 (32)	7 (2)
	**Control**	351	56 ± 10.7	387 (48.4)	34 (9.7)	NM	43 (12.3)	147 (41.9)	77.9 ± 11.5	107 (30)	3 (0.9)

Data are presented in mean±SD or proportions as (%). No: Number; SD: standard deviation; LDTC: Low-dose triple combination; BMI: body mass index; TTT: treatment; CKD: chronic kidney disease; NM: not mentioned.

Supplementary Table 5 Additional Baseline characteristics of the participants.

Study ID	Arms	Baseline automated office BP, mmHg		Baseline home BP, mmHg		Sodium, mmol/L	Potassium, mmol/L	Creatinine level, mg/dL	eGFR, c mL/min/1.73 m2
		Systolic BP	Diastolic BP	Systolic BP	Diastolic BP				
**Rodgers et al., 2024**	**LDTC**	137.5 ± 11.5	85.5 ± 9	138.5 ± 6.6	86 ± 7	NM	NM	NM	NM
	**Control**	139 ± 10	87 ± 9	139 ± 7	86 ± 9	NM	NM	NM	NM
**Ojji et al., 2024**	**LDTC**	156 ± 12	97 ± 8	150 ± 17	97 ± 11	143 ± 3	4.1 ± 0.5	0.9 ± 0.2	87 ± 20
	**Control**	156 ± 12	97 ± 8	151 ± 16	96 ± 11	143 ± 3	4.1 ± 0.4	0.9 ± 0.2	88 ± 19
**Sung et al., 2022**	**LDTC**	151.5 ± 11.8	91.3 ± 8.9	NM	NM	NM	NM	NM	NM
	**Control**	150.1 ± 10.6	92 ± 9	NM	NM	NM	NM	NM	NM
**Hong et al., 2020**	**LDTC**	154.5 ± 9.6	91.8 ± 8.7	NM	NM	141 ± 2.1	4.37 ± 0.4	0.9 ± 0.177	NM
	**Control**	154 ± 9	90.7 ± 9	NM	NM	140.9 ± 1.9	4.36 ± 0.37	0.89 ± 0.19	NM
**Webster et al., 2018 (TRIUMPH)**	**LDTC**	154.2 ± 11.3	89.5 ± 9.7	NM	NM	NM	NM	0.88 ± 0.87	90.1 ± 19.7
	**Control**	154.2 ± 11.6	90.0 ± 9.7	NM	NM	NM	NM	0.82 ± 0.29	91.8 ± 18.6

All data are presented in mean±SD format. M: Mean; LDTC: Low-dose triple combination; BP: blood pressure; NM: Not mentioned; eGFR: estimated glomerular filtration rate.

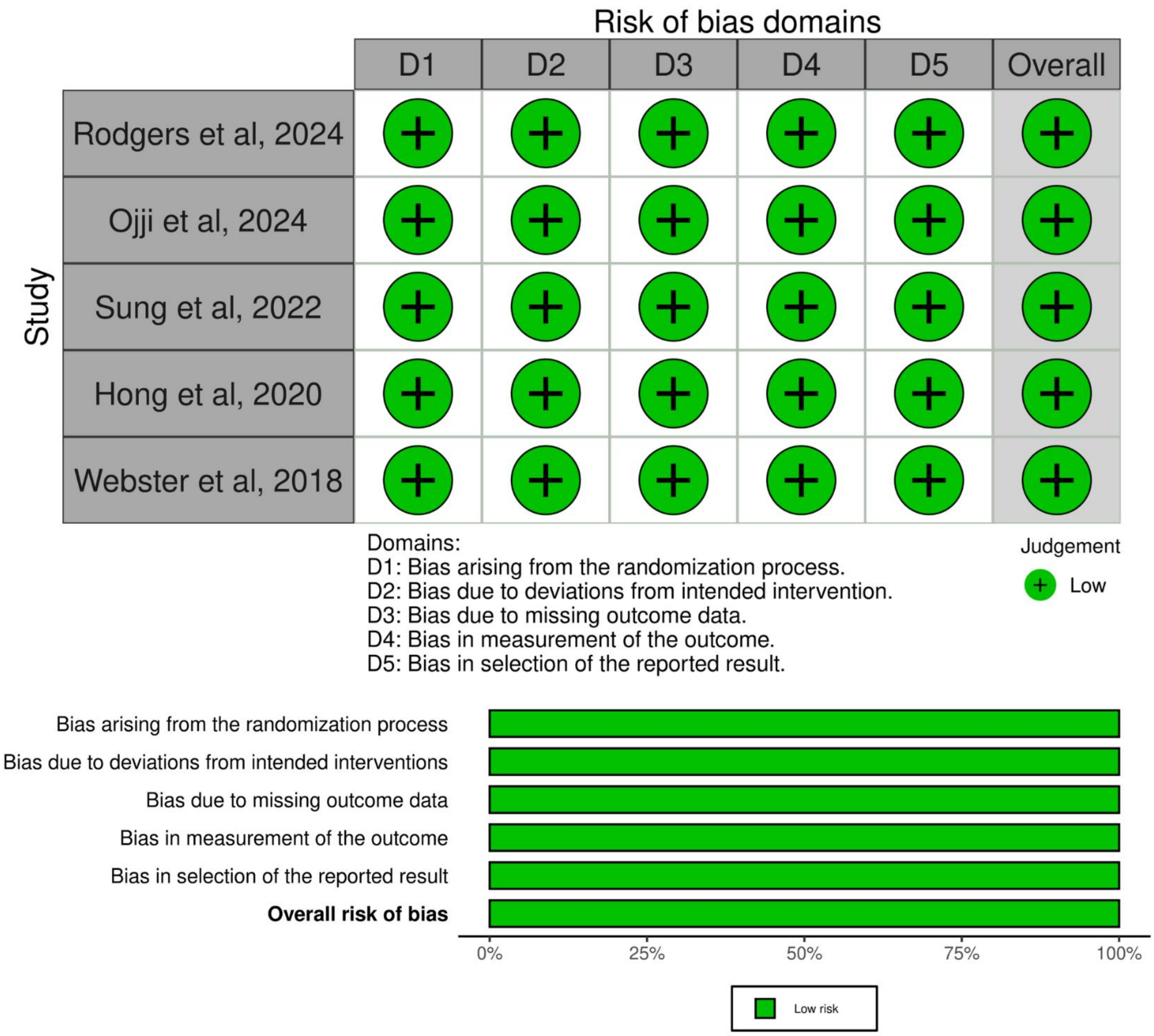

Supplementary Fig. 1 Overview of the risk of bias of the included randomized controlled trials.

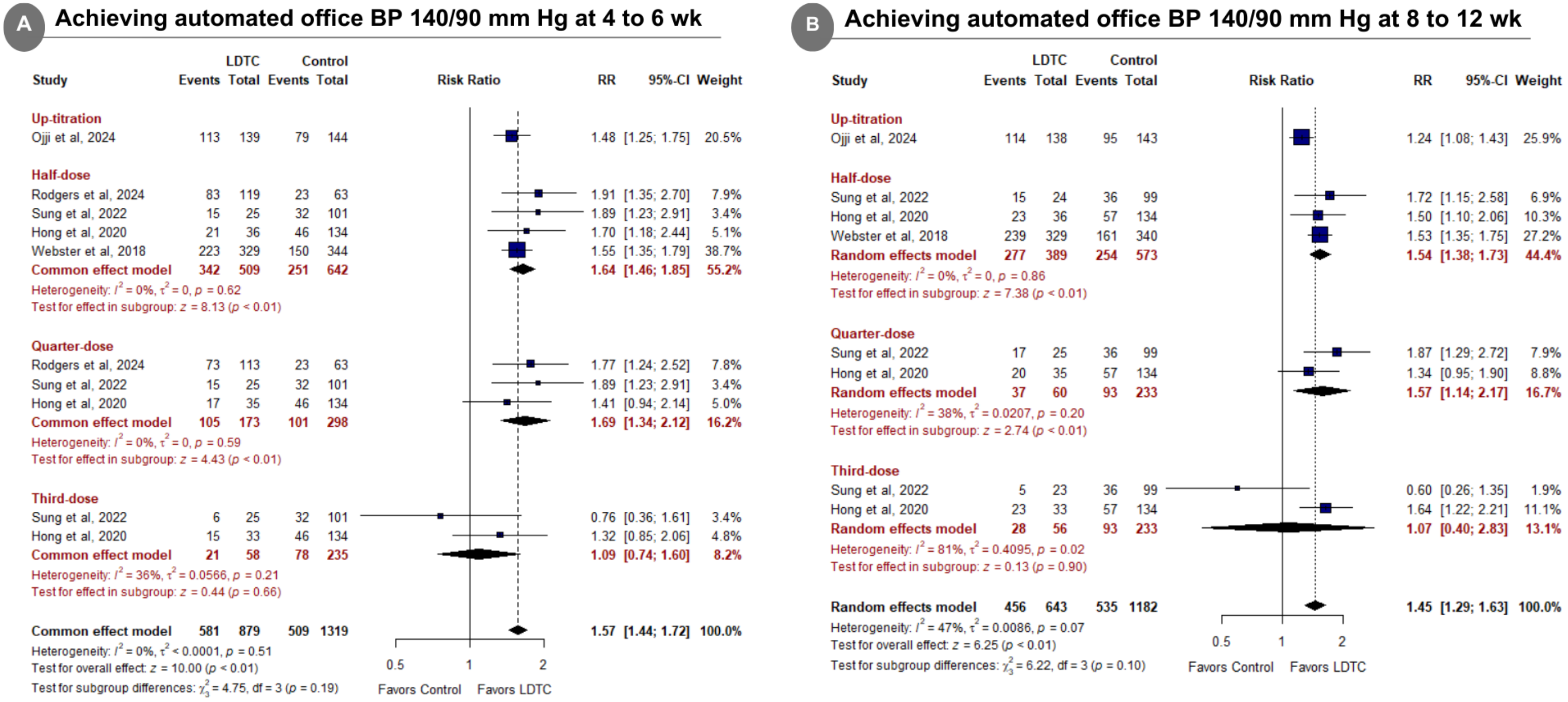

Supplementary Fig. 2 Forest plot of dosage subgroup analysis for achieving target automated office blood pressure control. *BP* blood pressure, *LDTC* low-dose triple combination, *RR* risk ratio, *CI* confidence interval.

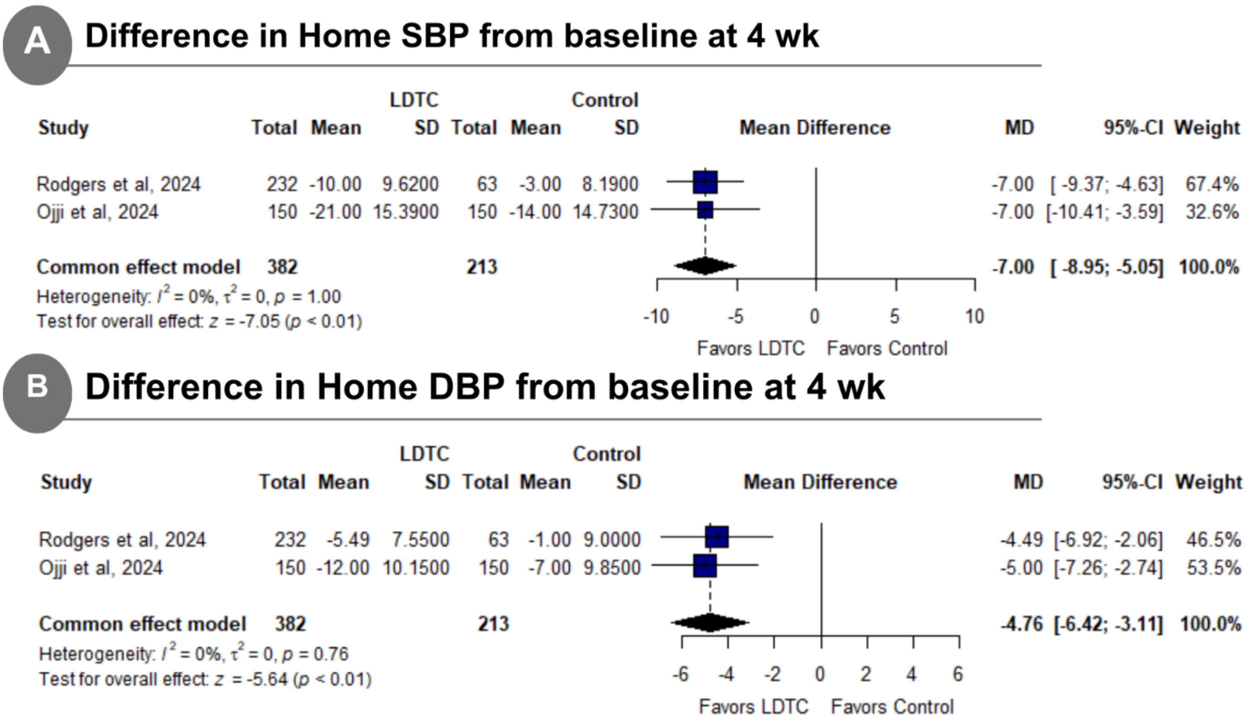

Supplementary Fig. 3 Forest plot of difference in home systolic and diastolic blood pressure at 4 weeks. *SBP* systolic blood pressure, *DBP* diastolic blood pressure, *LDTC* low-dose triple combination, *MD* mean difference, *CI* confidence interval.

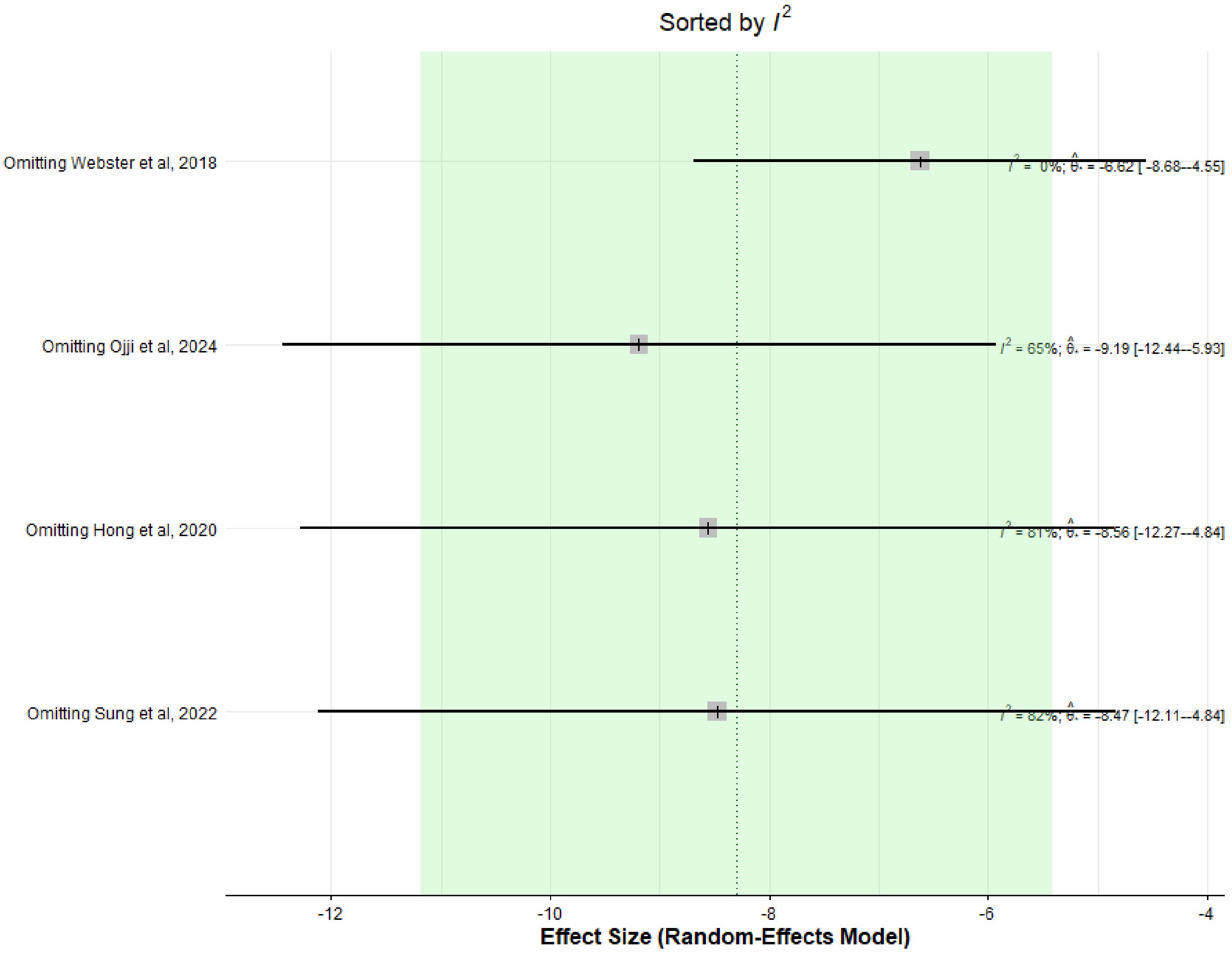

Supplementary Fig. 4 Leave-one-out sensitivity analysis of automated office systolic blood pressure from baseline at 8 to 12 weeks.

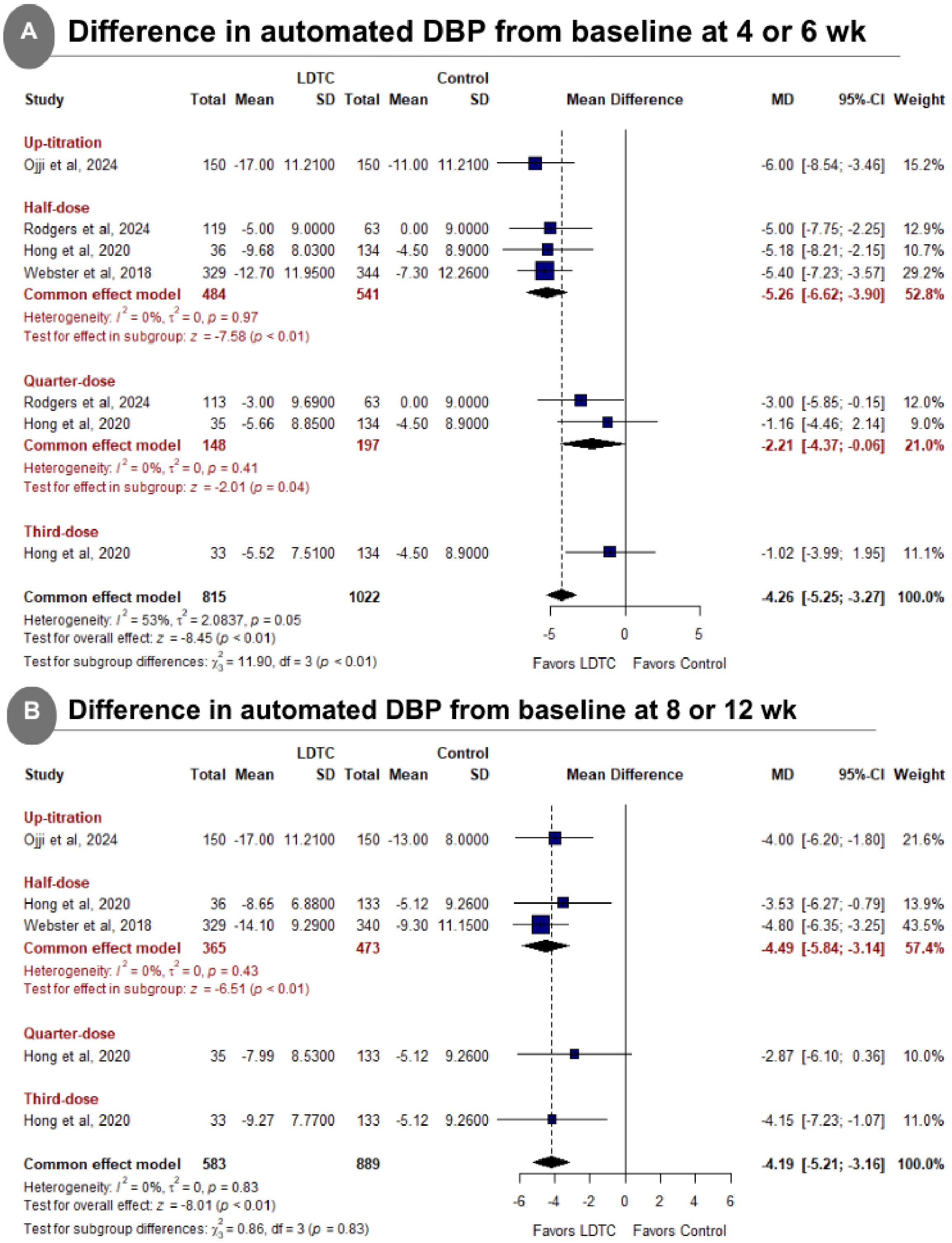

Supplementary Fig. 5 Forest plot of dosage subgroup analysis for difference in automated diastolic blood pressure. *DBP* diastolic blood pressure, *LDTC* low-dose triple combination, *MD* mean difference, *CI* confidence interval.

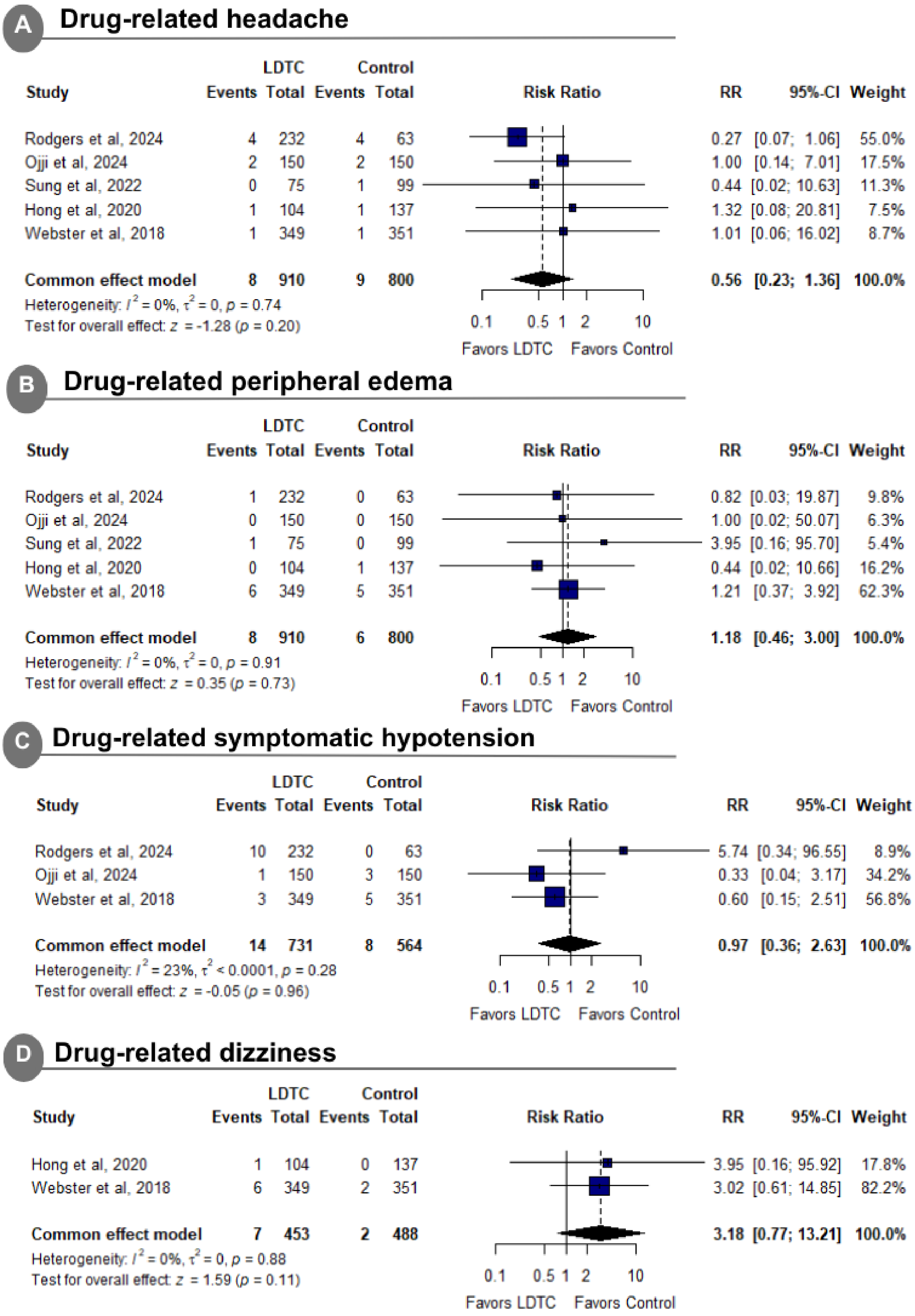

Supplementary Fig. 6 Forest plot of drug-related adverse events (headache, peripheral edema, symptomatic hypotension, and dizziness). *LDTC* low-dose triple combination, *RR* risk ratio, *CI* confidence interval.

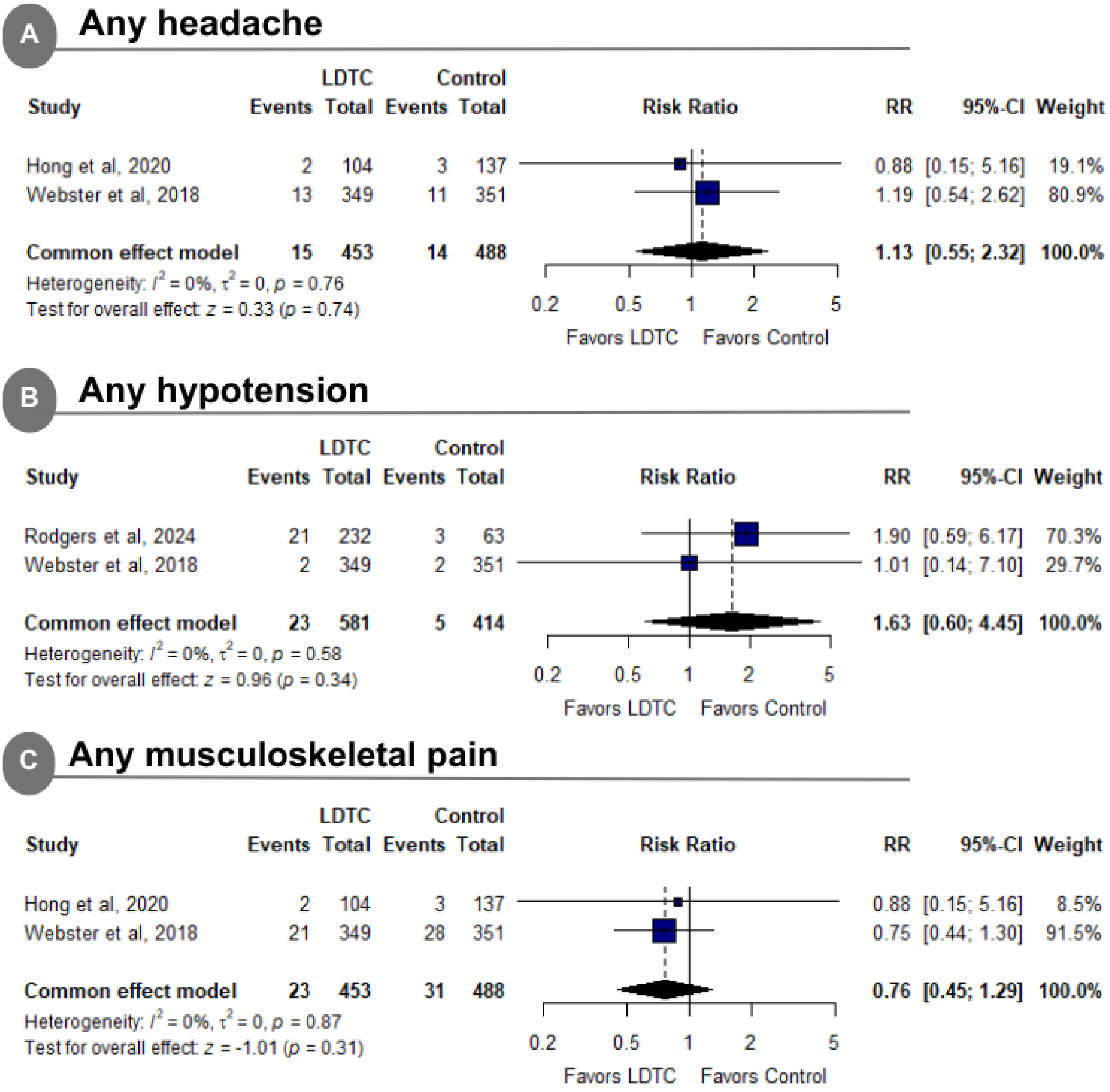

Supplementary Fig. 7 Forest plot of adverse effects for any reason. Any defined as the adverse event occurred in the patients for any reason across the study period. *LDTC* low-dose triple combination, *RR* risk ratio, *CI* confidence interval.

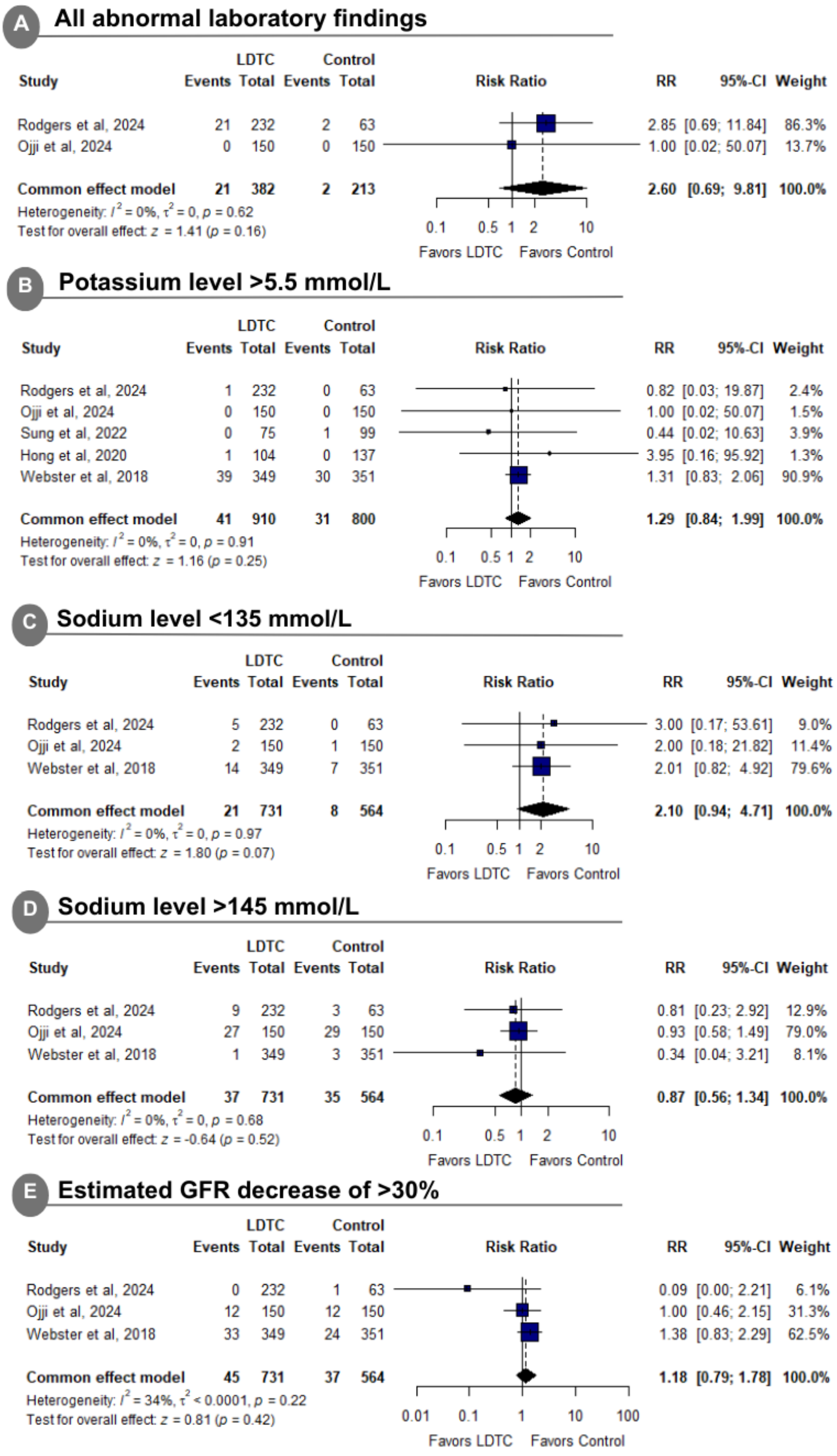

Supplementary Fig. 8 Forest plot of abnormal laboratory findings. *GFR* glomerular filtration rate, *LDTC* low-dose triple combination, *RR* risk ratio, *CI* confidence interval.

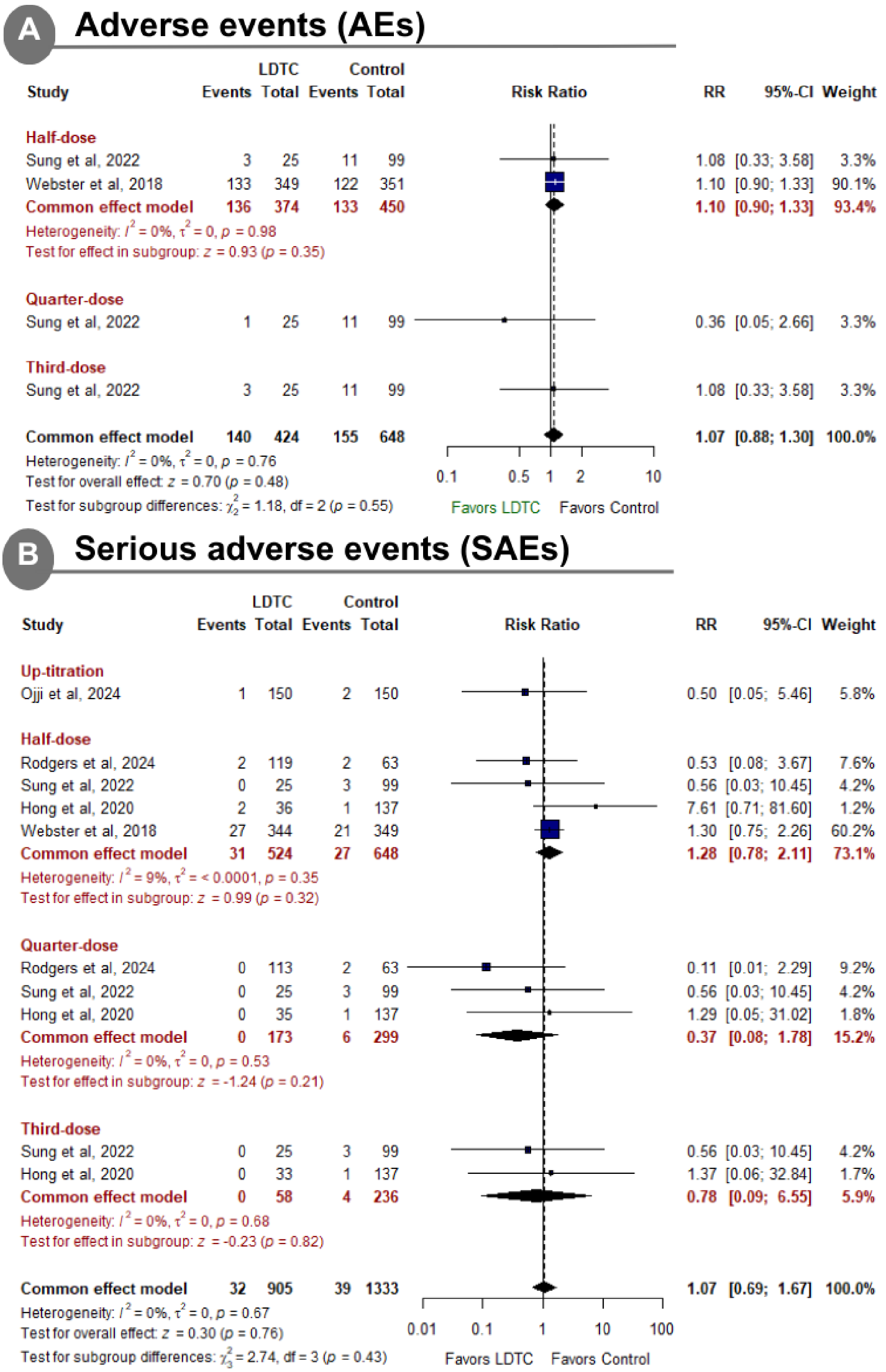

Supplementary Fig. 9 Forest plot of dosage subgroup analysis for adverse events and serious adverse events. *LDTC* low-dose triple combination, *RR* risk ratio, *CI* confidence interval.

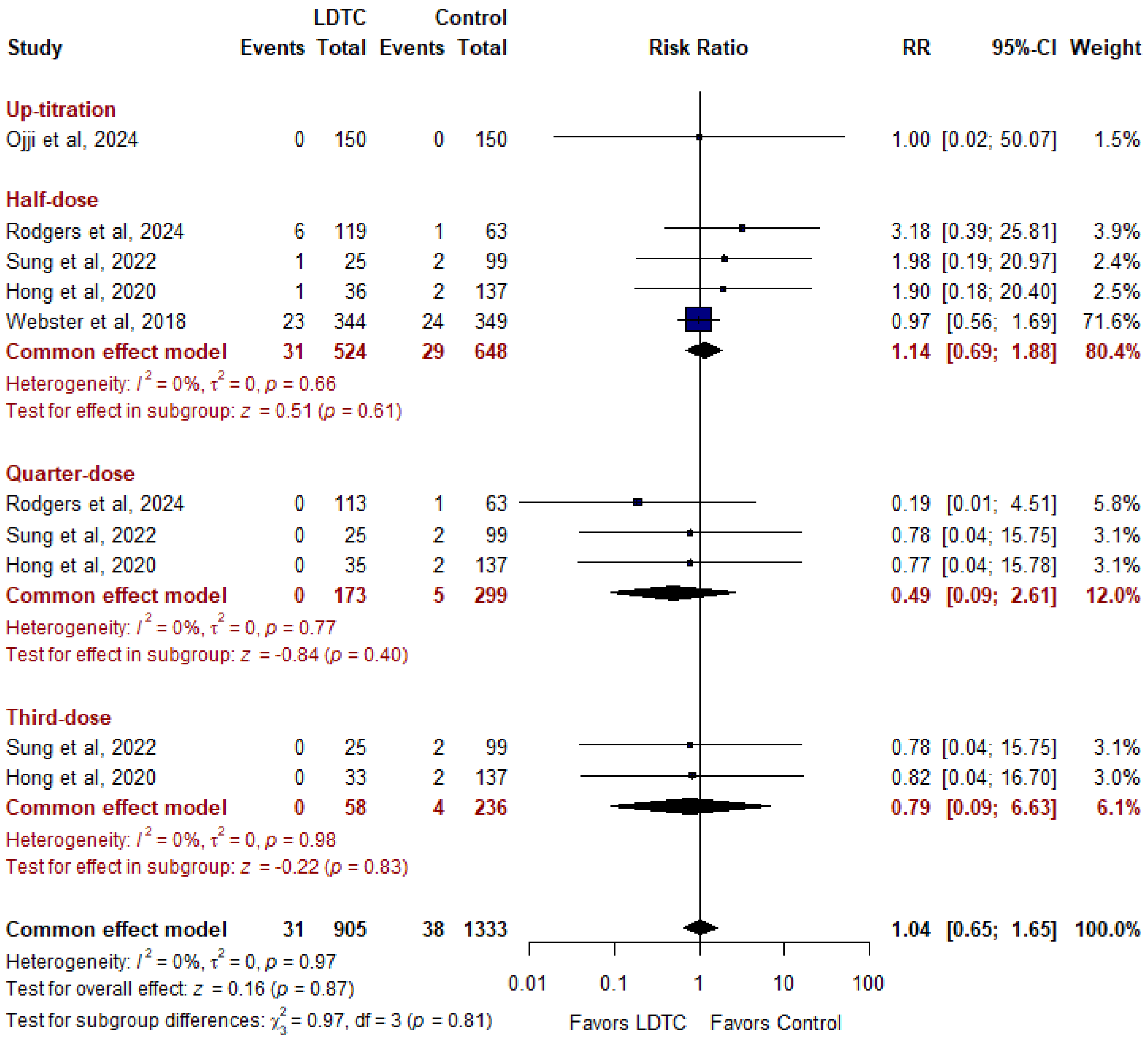

Supplementary Fig. 10 Forest plot of dosage subgroup analysis for treatment discontinuation due to adverse events. *LDTC* low-dose triple combination, *RR* risk ratio, *CI* confidence interval.

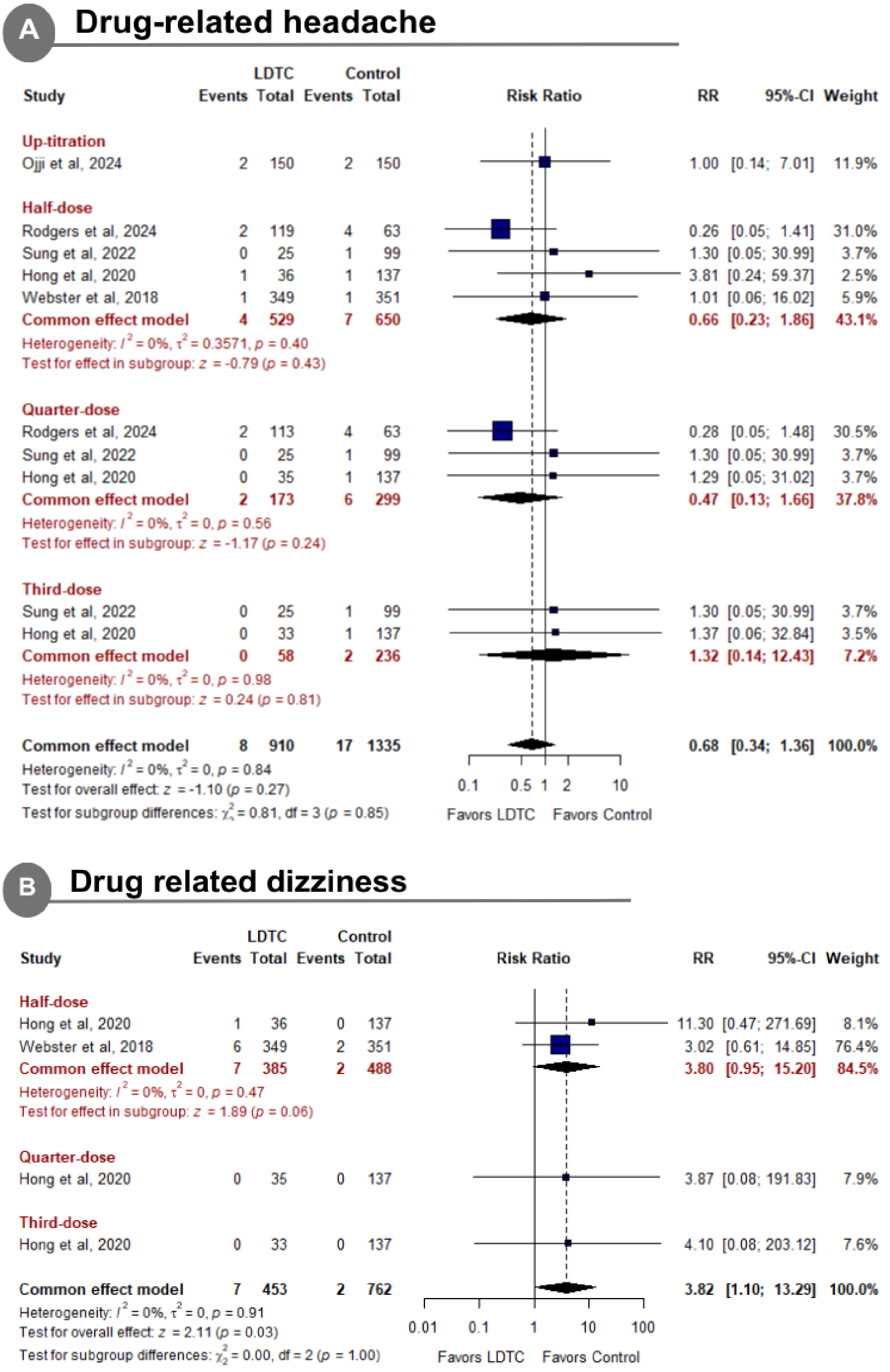

Supplementary Fig. 11 Forest plot of dosage subgroup analysis for drug-related adverse events (headache and dizziness). *LDTC* low-dose triple combination, *RR* risk ratio, *CI* confidence interval.

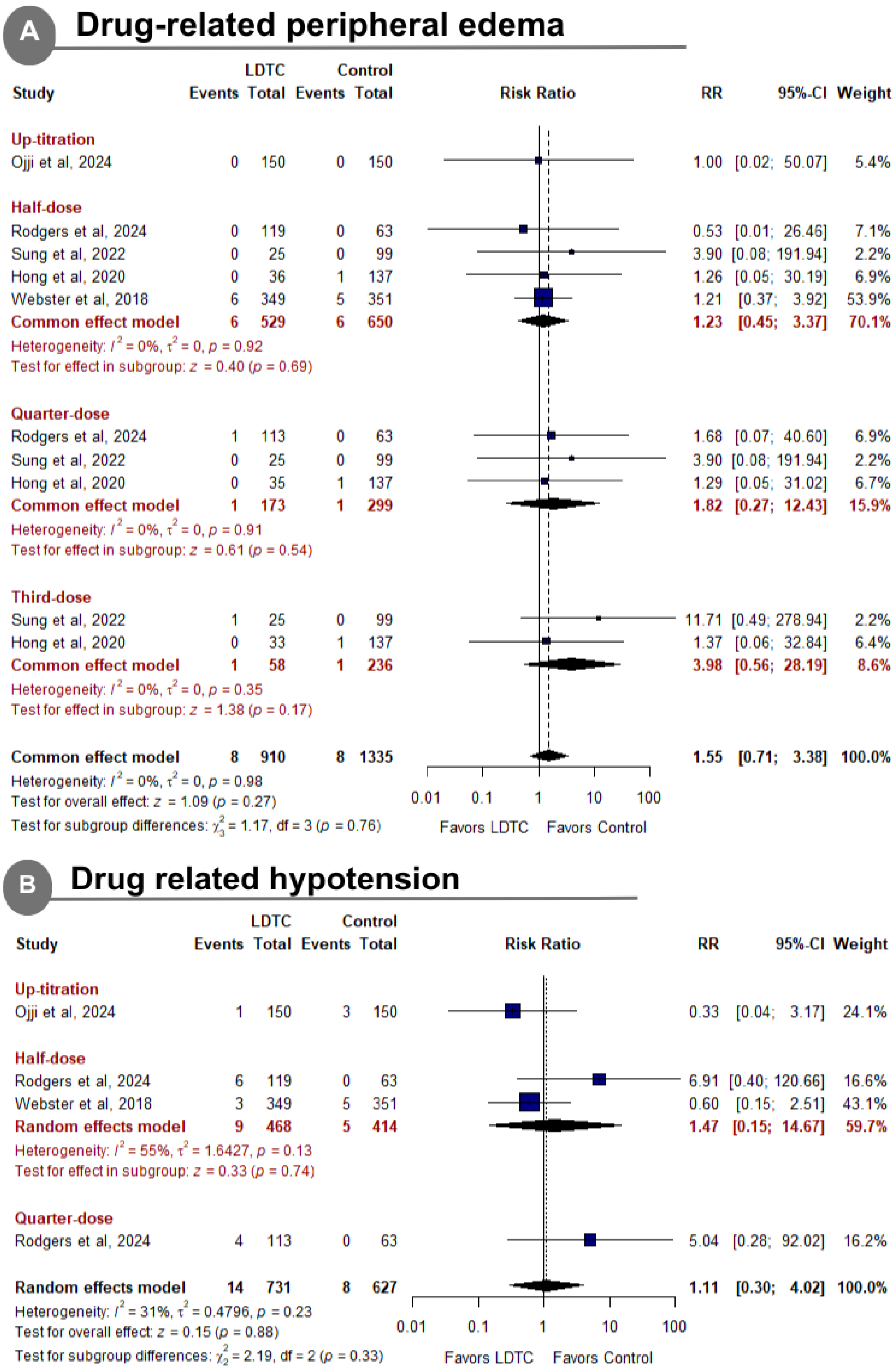

Supplementary Fig. 12 Forest plot of dosage subgroup analysis for drug-related adverse events (peripheral edema and hypotension). *LDTC* low-dose triple combination, *RR* risk ratio, *CI* confidence interval.

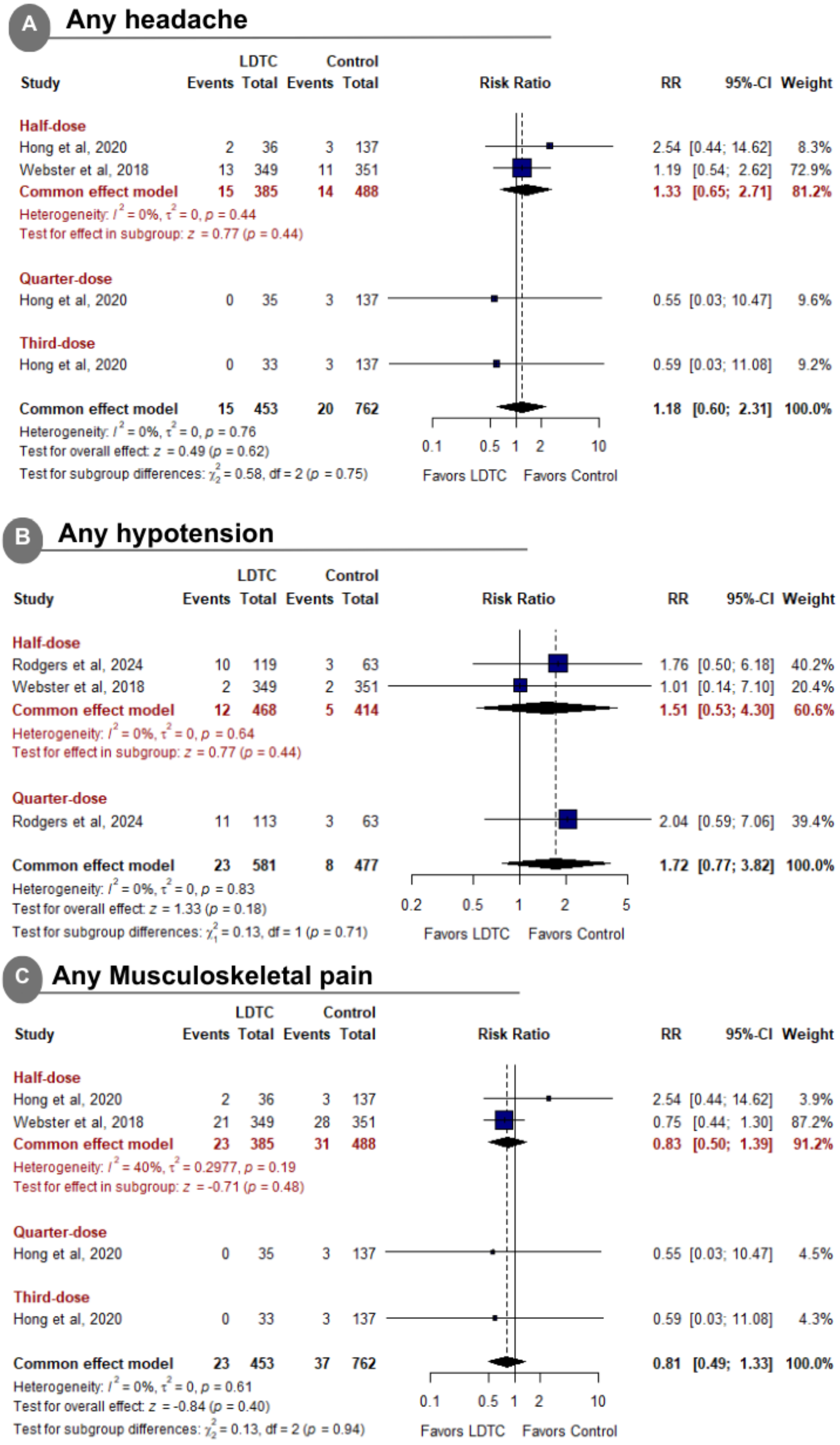

Supplementary Fig. 13 Forest plot of dosage subgroup analysis for adverse effects for any reason. Any defined as the adverse event occurred in the patients for any reason during the study period. *LDTC* low-dose triple combination, *RR* risk ratio, *CI* confidence interval.

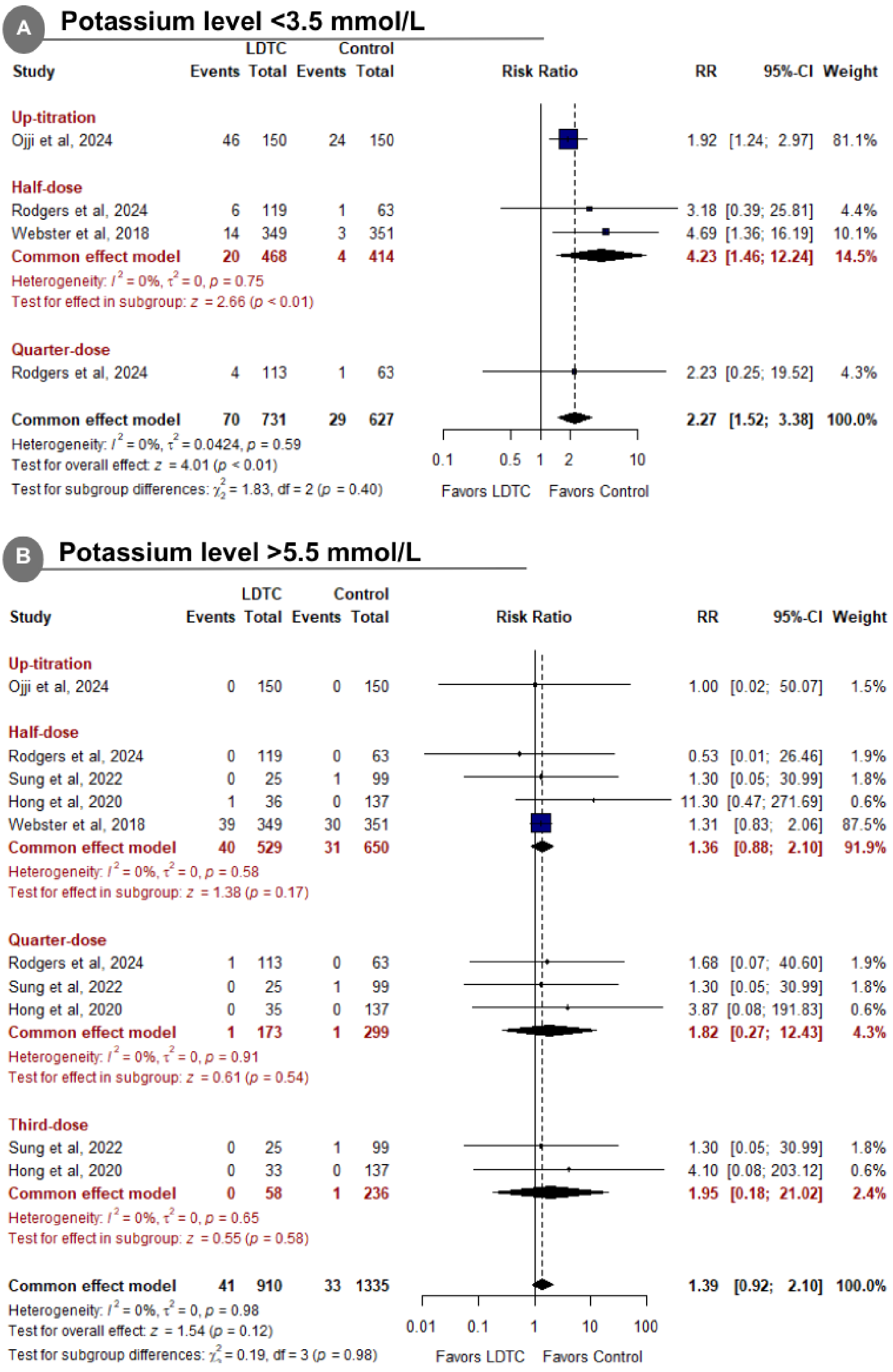

Supplementary Fig. 14 Forest plot of dosage subgroup analysis for decreased and increased potassium levels. *LDTC* low-dose triple combination, *RR* risk ratio, *CI* confidence interval.

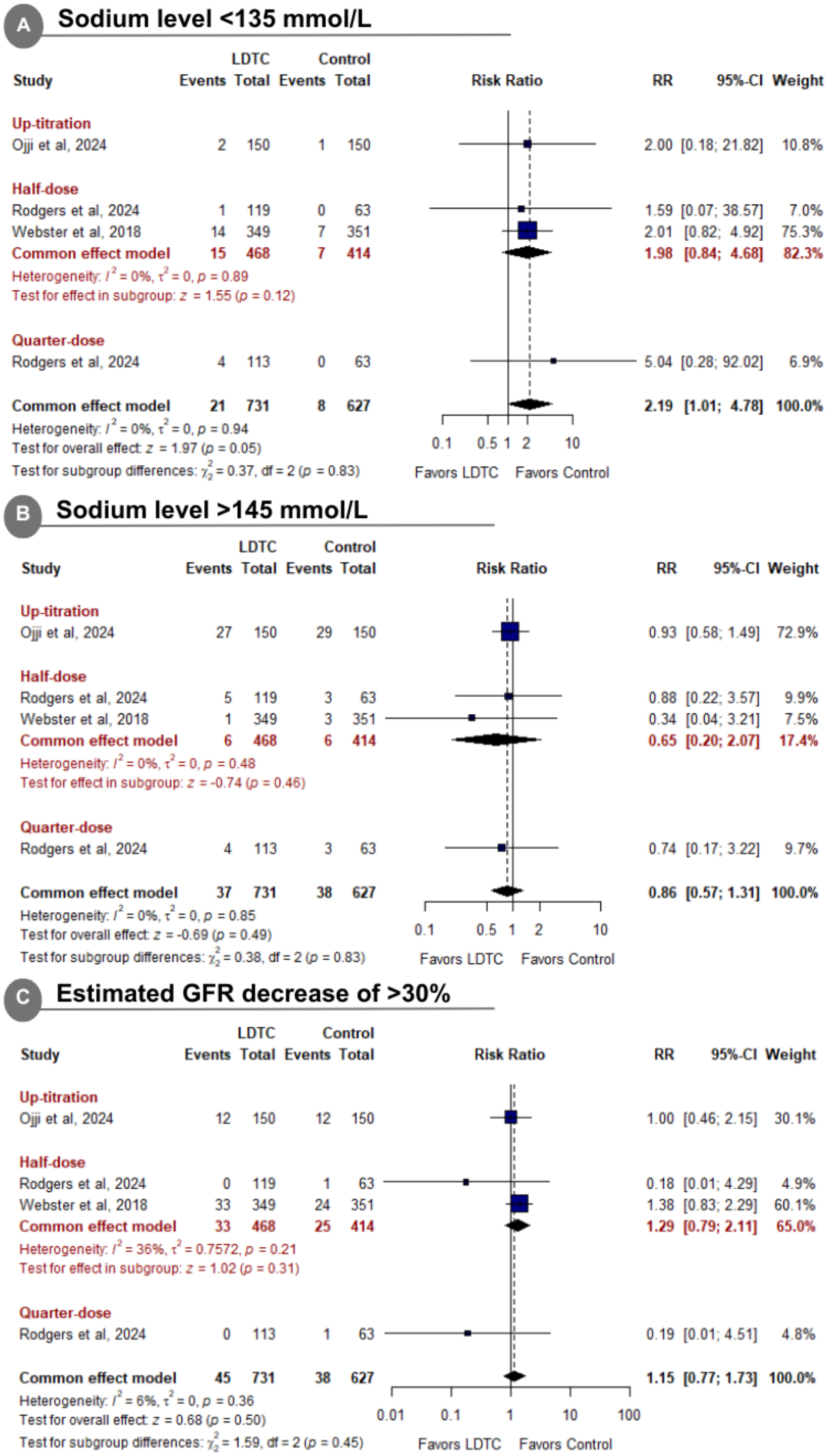

Supplementary Fig.15 Forest plot of dosage subgroup analysis for decreased and increased sodium levels and decreased estimated glomerular filtration rate. *GFR* glomerular filtration rate *LDTC* low-dose triple combination, *RR* risk ratio, *CI* confidence interval.
